# Reversible Redox‐Responsive Peptide Condensates With RNA

**DOI:** 10.1002/cbic.70513

**Published:** 2026-08-21

**Authors:** Anika L. Moller, Pall Thordarson

**Affiliations:** ^1^ School of Chemistry and the UNSW RNA Institute University of New South Wales Sydney New South Wales Australia

**Keywords:** condensates, dynamic covalent chemistry, peptides, redox‐responsive, RNA

## Abstract

While the amino acid cysteine is usually an “order promoting” amino acid, not normally found in disordered proteins which undergo phase separation, researchers have found cysteine‐rich regions flanking low‐complexity domains, imparting redox sensitivity to condensates both in vivo and in vitro. However, these examples of redox reversible condensates in vitro lose their reversibility when RNA (an integral component of biomolecular condensates) is added. Herein, we demonstrate a system where a cationic peptide containing a cysteine residue forms redox reversible condensates with RNA. Additionally, this peptide formed condensates with RNA preferentially over the DNA analogs of the sequence, demonstrating the importance of RNA in this system. Fluorescence recovery experiments determined that the condensates displayed similar viscoelastic properties after dissolution and reformation in this manner, and finally a control peptide lacking a cysteine did not form condensates under oxidizing conditions.

## Graphical abstract

Here, we demonstrate a system derived from biologically relevant peptide and RNA oligomers, which form redox‐switchable condensates that are formed and dissolved via a cysteine handle on the peptide.

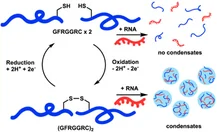

## 1 Introduction

The disulfide “bridge” formed between two thiols is an example of dynamic covalent chemistry (DCC) which is heavily used by biology and scientists alike. In nature, the amino acid cysteine is one of the least abundant but is over‐represented in functional sites on proteins (e.g., catalytic sites and cofactor binding) [1]. Cysteine is also believed to be one of the latest evolving amino acids and is over‐represented in more complex eukaryotic organisms, likely due to its ability to impart additional complex structure and function to proteins [2, 3]. Cysteine is the only amino acid with a thiol group, which lends it to participating in a number of interesting chemistries, namely, the ability to undergo controllable redox reactions in relatively mild conditions. Cysteines are often seen as “order promoting” amino acids, as the disulfide bridges formed either intra‐ or intermolecularly are utilized to fold proteins in specific ways or to connect multiple subunits together. Being able to reversibly switch between the reduced, free thiol form and the oxidized, disulfide form, cysteine residues in proteins are able to respond to certain cellular stimuli such as oxidative stress and regulate signals [4].

Biomolecular condensates are membraneless cellular components which are typically comprised of intrinsically disordered proteins that undergo liquid–liquid phase separation (LLPS) [5, 6, 7]. These proteins contain regions of amino acids which impart low complexity. As mentioned, cysteine is an order‐promoting amino acid and hence is under‐represented in intrinsically disordered proteins [8]. However, this does not mean that cysteine should be ignored as an important amino acid in biological condensates, as a closer look into the human proteome has found numerous cysteine‐rich sections flanking low‐complexity domains which may impart redox sensitivity to these proteins [4]. Biomolecular condensates are implicated in being responsible for numerous functions in the cell and are now being utilized by researchers in the development of dynamic, soft materials or as delivery mechanisms [9, 10, 11, 12]. These condensates utilize the many classical noncovalent interactions such as electrostatic, cation–*π* and *π*–*π* in their formation, where dynamicity can be afforded by changes in pH or ionic strength. However, there is little research into how the redox‐sensitive properties of thiol motifs, such as in cysteine, may form dynamic condensates.

A simple peptide‐based synthon developed by Abbas et al. has been shown to be redox sensitive by the inclusion of a disulfide bridge [13]. In this case, the oxidized, disulfide form of the synthon would undergo self‐coacervation and, when reduced to the free thiol form, would dissolve. In another example of “unnatural” redox, reversible condensates, Chowdhuri et al. utilized two different DCC “levers,” the redox‐responsive disulfide bond and the pH‐responsive imine [14]. This system would only undergo LLPS when both of these dynamic covalent bonds were formed, and hence, two different triggers could be modulated in the formation of condensates. This pH dependency enabled the researchers to create a pH clock with the system, as when kept in the oxidized, disulfide form, transient condensates that would form and dissolve with only the original trigger were made. Like with Abbas et al. [13], the species undergoing phase separation, while bioinspired, were still synthetic molecules not found in the cell. However, this does not mean that these motifs are not potentially responsible for the formation of membraneless organelles, including with RNA in multicomponent condensates.

**FIGURE 1:**
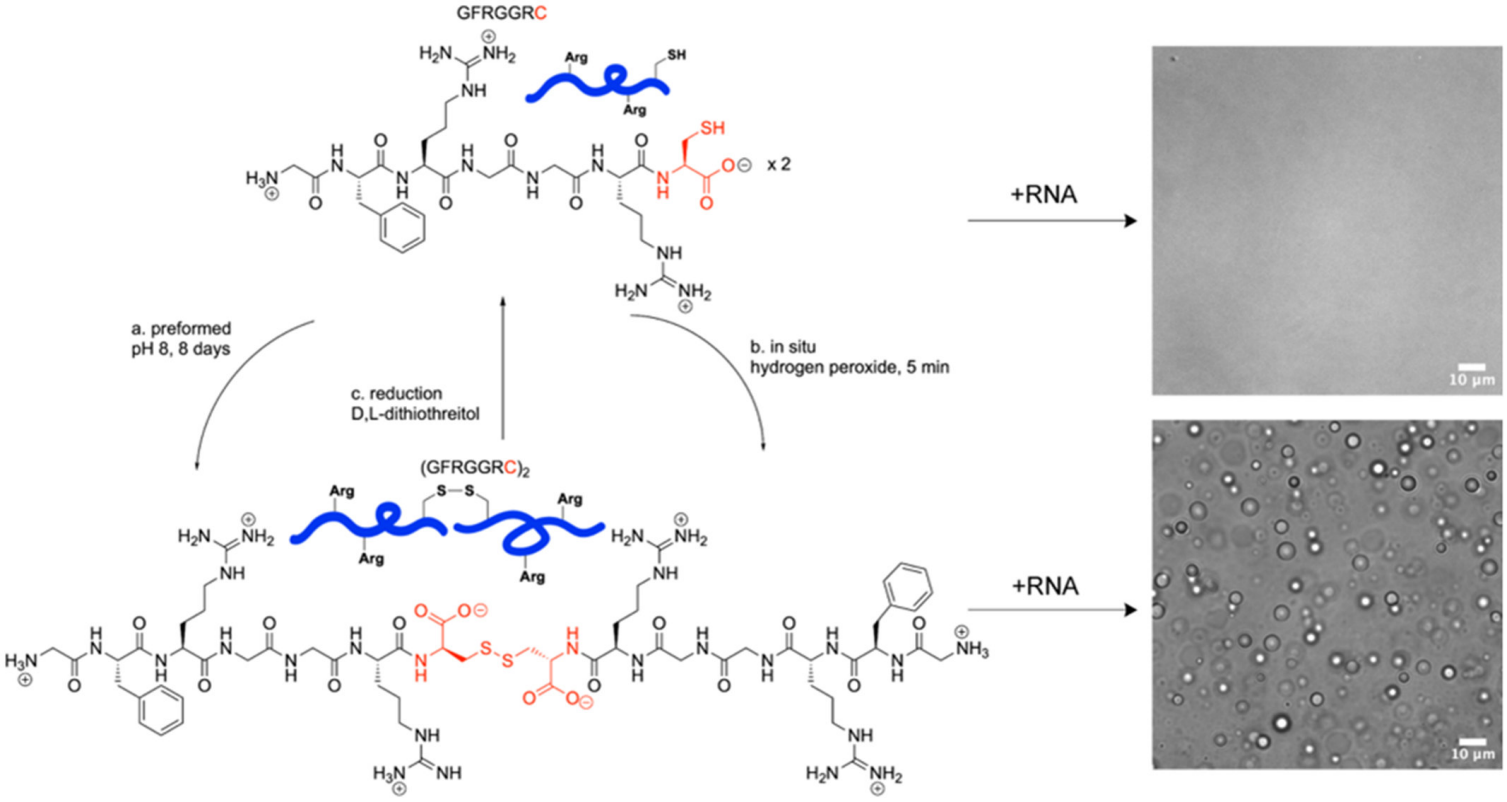
Schematic describing the two ways in which the dimer **(GFRGGRC)**_**2**_ was formed in this work, (a) gently, by slow oxidation in slightly basic buffer, ammonium carbonate (pH 8, 0.01 M) followed by HPLC purification and characterization, and (b) quickly by the addition of hydrogen peroxide. In both instances, the monomer **GFRGGRC** was regenerated by (c) reduction with d,l‐dithiothreitol. Brightfield microscopy images of these peptides combined with RNAs are shown on the left.

Bhopatkar et al. have found that the usually order‐promoting amino acid, cysteine (which contains a thiol motif that can form reversible disulfide bridges), is found to be flanking low‐complexity domains of the protein TDP‐43 [15]. TDP‐43 is a protein which undergoes phase separation with RNA in the cytoplasm during times of cellular stress, and the subsequent formation of solid aggregates is implicated in ALS disease progression. They demonstrated that the TDP‐43 protein and RNA system’s dynamics could be modulated between under redox gradients, where crosslinking within or between these flanking cysteine‐rich domains would increase the viscosity of the condensates, sometimes forming gels or solid aggregates [4, 16]. From the same research group, Mondal et al. were able to create an in vitro model based on the TDP‐43 protein, where 15‐mer RGG‐repeat‐based peptides were shown to undergo segregative LLPS at lower concentrations when cysteine residues were incorporated under oxidizing conditions [17]. These condensates could then be dissolved by the addition of reducing agent d,l‐dithiothreitol (DTT), and reformed upon the addition of the oxidizing agent hydrogen peroxide. However, these condensates were not shown to dissolve and reform with multiple cycles, and once RNA was added, this redox sensitivity was lost, likely due to the RGG‐repeat‐rich peptides being able to readily undergo LLPS with the RNA even in their reduced, free thiol form. Hence, a fully reversible system which contains associative phase separation with RNA and remains redox sensitive is yet to be developed. We report here such a system based on a cationic peptide **GFRGGRC** containing a containing a cysteine residue forms redox reversible condensates with RNA (Figure 1).

## 2 Results and Discussion

### 2.1 Design of a Redox‐Responsive RNA‐Peptide Condensate

In order to design a complex coacervation (i.e*.*, where condensates form between two polyelectrolytes) system that is redox reversible, either one or both molecules must form condensates in the oxidized or reduced form, but not in the other. In this study, the peptide was the obvious choice to make as the redox sensitive component, as the thiol‐containing amino acid cysteine can be easily incorporated into any peptide and readily forms a disulfide bond with itself under oxidizing conditions. Based on previous work in our group, it was determined that at neutral pH when combined with short decameric (10‐mer) (**adenosine‐guanidine**)_**5**_ **[(AG)**_**5**_ **]** RNA, the short RGG‐repeat‐based peptides would not from condensates if it had less than three arginine residues (at least at the tested concentrations ≤5 mg mL^−1^ each) [18]. Hence, the peptide sequence **GFRGGRC** was chosen, as it contains only two arginines and would therefore be unlikely to form condensates with a decameric RNA. The phenylalanine residues were included to allow the peptide to interact with the RNA by aromatic modes.

The C‐terminal cysteine residue incorporated was included as the redox active component. Once the peptide is oxidized, it should form a disulfide bond between two of itself, forming a dimer peptide which now contains four arginines. This longer peptide should form condensates with a decameric RNA in solution. The control peptide **GFRGGRS** was also synthesized where the cysteine has been mutated to a serine, which is a close mimic but with an alcohol group rather than a thiol. Therefore, this peptide should not be oxidizable into a cystine dimer and not form condensates with the RNA in solution even under oxidizing conditions.

The oxidation of the peptide, **GFRGGRC**, into the dimer, **(GFRGGRC)**_**2**_, was performed in two ways (Figure 1). First, the dimer was *preformed* before phase separation experiments with **(AG)**_**5**_ RNA were undertaken. This was done under gentle oxidizing conditions to form the disulfide cleanly (i.e., without further oxidation products) and to allow for more extensive characterization. In addition, this was done for experiments with **(AG)**_**5**_ to confirm that the emergent properties seen in the experiment were in fact due to a dimer forming, rather than some other effect caused by the addition of the oxidizing agent. Second, the oxidizing agent hydrogen peroxide (H_2_O_2_) was used to form the dimer in situ.

**FIGURE 2:**
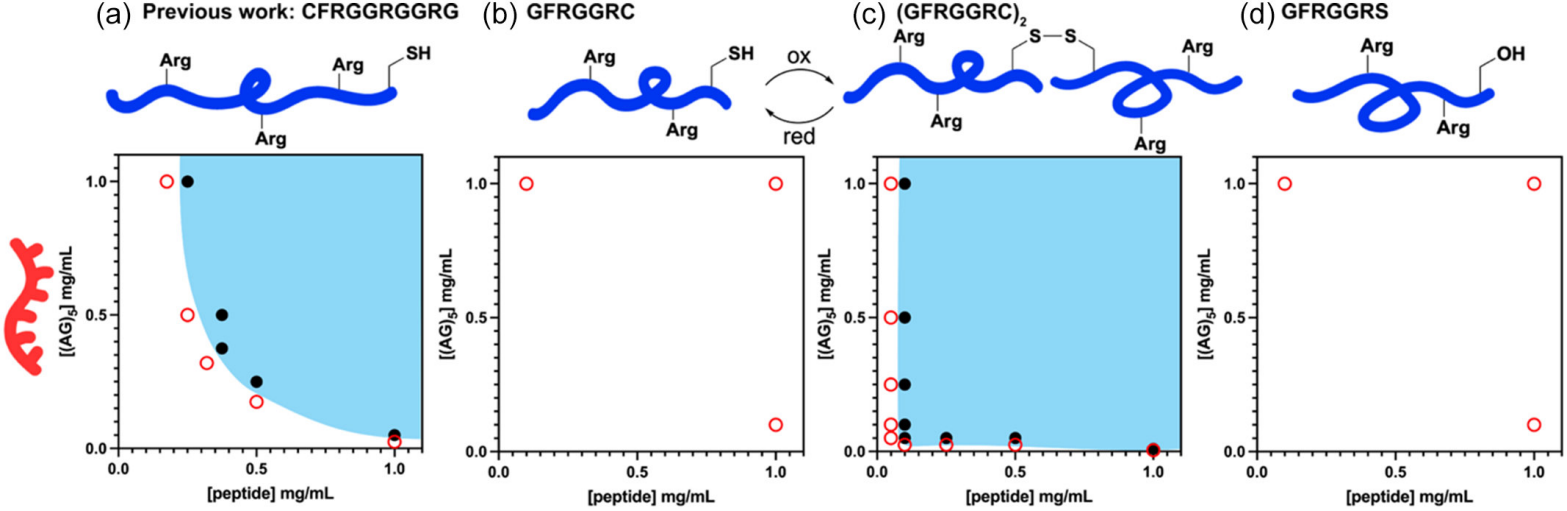
Phase diagrams of the peptides (a) **CFRGGRGGRG** (from previous work [18]), (b) monomer **GFRGGRC**, (c) dimer **(GFRGGRC)**_**2**_, and (d) control **GFRGGRS** as combined with decameric **(AG)**_**5**_ RNA up to 1 mg mL^−1^ of each in sodium phosphate buffer (pH 7, 0.1 M). Red open circle represents the absence of condensates, and black closed circles represents the presence of condensates, based on turbidity measurements above 0.1 at 450 nm.

The simple RNA decamer **(AG)**_**5**_ was used in this investigation, as it has been shown by previous work in the group to readily form condensates with RGG‐repeat peptides and does not form any kind of secondary structure [18]. From the phase diagram, it is clear that monomer **GFRGGRC** does not form condensates in the tested range of concentrations, while oxidized dimer **(GFRGGRC)**_**2**_ does at very low concentrations of both RNA and peptide (Figure 2). Notably, at excess peptide concentration, very low quantities of the decameric **(AG)**_**5**_ RNA (1 and 0.01 mg mL^−1^, respectively) are required for LLPS to occur. Furthermore, neither peptides nor RNA phase separate themselves, even under oxidizing conditions (no turbidity measured at 450 nm as per Figure S20). Therefore, we have demonstrated a system which undergoes LLPS when in the oxidized form with RNA, but not the reduced.

To reduce the peptide back into **GFRGGRC**, DTT was determined to be the most appropriate reducing agent (Figure S21) [17, 19]. Upon the addition of DTT, the condensates would dissolve by turbidity measurements and microscopy. While the initial formation of condensates is simple to observe with a single turbidity measurement immediately upon mixing, it can become more difficult when one observes changes in turbidity over time. As seen from the control data without polyethylene glycol 8000 (PEG‐8000) in Figure S22, the decrease in turbidity upon the addition of reducing agent was too similar to the decrease due to aging and hence was not a reliable indicator that reduction and dissolution of the condensates had occurred. Since it has been determined that the presence of longer PEGs in solution stabilizes the condensate “emulsion” and keeps the solution turbid, PEG‐8000 (10%, w/v) was added to rectify this in all future experiments [18].

The reduced **GFRGGRC** peptide and **(AG)**_**5**_ RNA decamer were combined with different concentrations of hydrogen peroxide. A turbidity increase was observed for 3 mM and above, corresponding to the formation of condensates, as confirmed by brightfield microscopy (Figure S23). This would indicate that not enough of the peptide is oxidized in situ when ≤2 mM hydrogen peroxide added, as the concentration of the peptide in solution is only around 1.3 mM. In addition, the stability of hydrogen peroxide solutions is very poor at low concentrations and nonacidic pH (above pH 4.5), so it is likely that the hydrogen peroxide has degraded into water and diatomic oxygen before being able to react with enough peptide to form condensates [20]. With 3 mM and higher concentrations of peroxide, the formation of condensates is due to sufficient **GFRGGRC** oxidation into the in situ **(GFRGGRC)**_**2**_ peptide and its interaction with **(AG)**_**5**_. As per the phase diagram, not much of the oxidized peptide (0.1 mg mL^−1^, or 10%) is needed to form condensates with 1 mg mL^−1^ of **(AG)**_**5**_ (Figure 2c). Therefore, it is likely that the oxidation reaction has not gone to completion and would explain why the turbidity level is not as high as when formed with the *preformed* **(GFRGGRC)**_**2**_ (Figures S22b vs. S23). To confirm that the formation of condensates is due to the oxidation of the cysteine residue, the same treatment was applied to a solution of the control peptide **GFRGGRS**, which, when combined with the RNA **(AG)**_**5**_ and 50 mM hydrogen peroxide, did not show any phase separation by turbidity measurement or microscopy (Figure S27).

### 2.2 Reversibility of Redox‐Responsive Condensate

**FIGURE 3:**
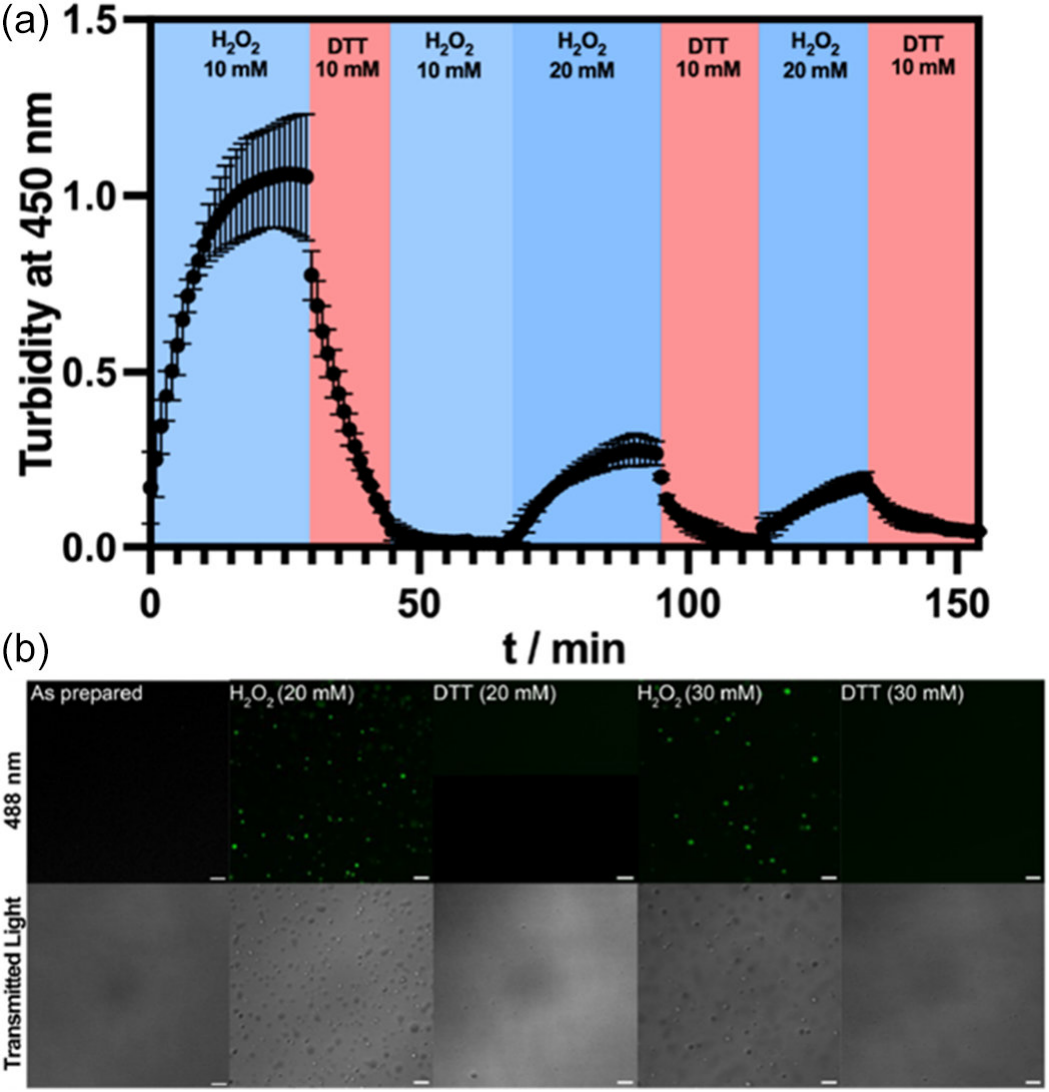
(a) Turbidity at 450 nm and (b) confocal microscopy images over time of condensate solutions formed with **GFRGGRC** (1 mg mL^−1^) and **(AG)**_**5**_ (1 mg mL^−1^) in sodium phosphate buffer (0.1 M, pH 7, PEG‐8000, 10%, w/v). H_2_O_2_ and DTT were added sequentially at indicated concentrations, indicated by blue and red shading, respectively, which should trigger the in situ formations of **(GFRGGRC)**_**2**_ and **GFRGGRC**, respectively.

Figure 3 shows the turbidity over time of the solution containing **GFRGGRC** and **(AG)**_**5**_, with sequential additions of hydrogen peroxide and DTT to form and dissolve the condensates reversibly. Initial incubation of the solution with 10 mM hydrogen peroxide shows an increase in turbidity over time, which sharply decreased upon the addition of 10 mM DTT. However, 10 mM hydrogen peroxide was not sufficient to reoxidize the peptide and reform the condensates, and 20 mM was needed to reform the condensates. 10 mM DTT was sufficient to reduce and dissolve the reformed condensates, which suggests that the hydrogen peroxide in solution does degrade quickly, but DTT remains in solution and competes with the peptide for the oxidation reaction, so more (20 mM) hydrogen peroxide is required for reoxidation.

This was repeated twice, with increasing concentrations of oxidizing and reducing agents each cycle, to ensure that excess DTT, for example, would not compete with the peptide in the oxidation reaction upon the addition of hydrogen peroxide. With each cycle, the maximum turbidity reached after 30‐min incubation decreased, with the maximum absorbance at 450 nm decreasing from ~1.0 to ~0.4 to ~0.3 (Figure 3a). This could be due to the excess DTT in solution competing in the oxidation reaction [21]. In addition, image analysis determined that the size of the condensates increased and the quantity in solution decreased, which could correlate to a lower turbidity measurement (Figure S24) [22]. Confocal microscopy with the addition of fluorescently labeled **GFRGGRC‐Fluor** was performed. Formation of condensates was seen upon the addition of hydrogen peroxide, and they would dissolve when DTT was added in a reversible manner (Figure 3b).

**FIGURE 4:**
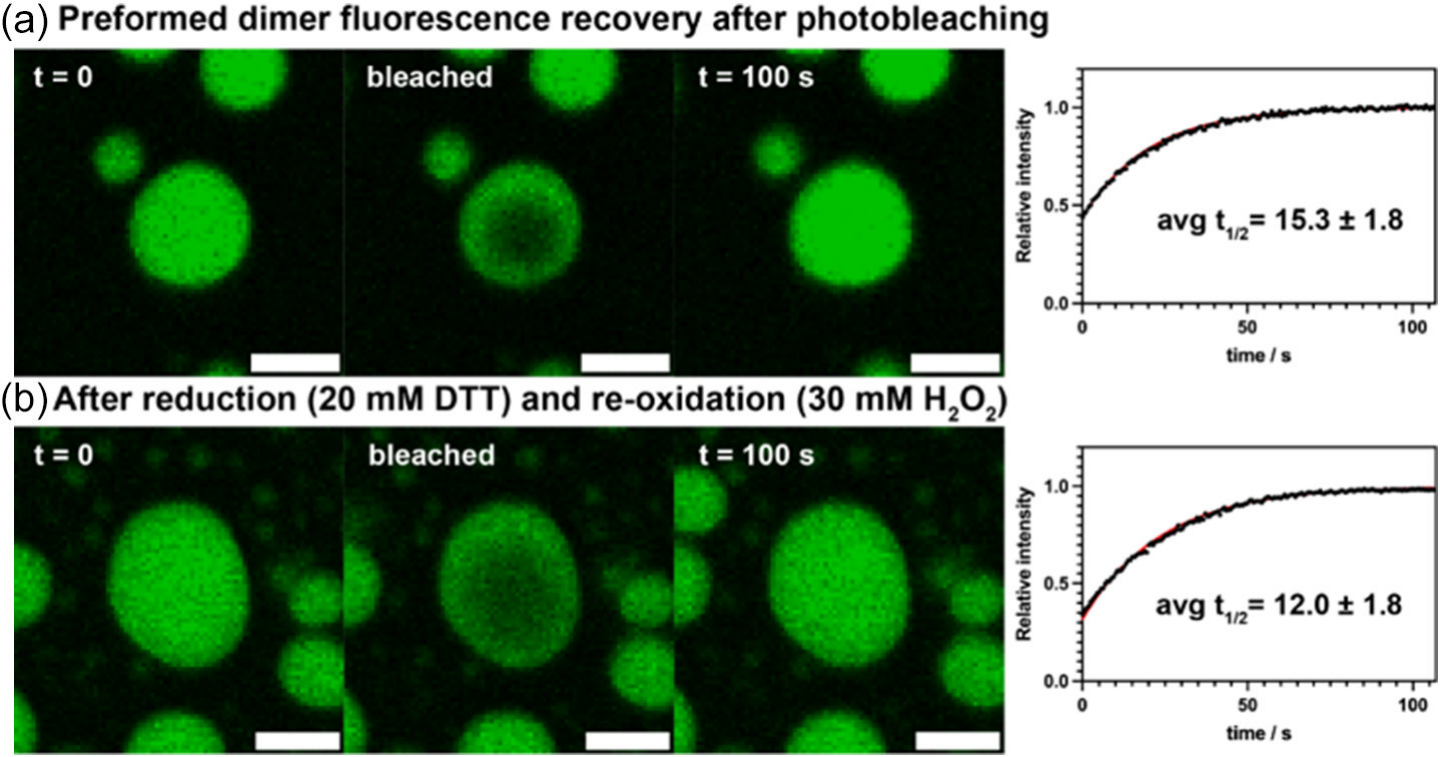
FRAP of condensates formed with (a) preformed **(GFRGGRC)**_**2**_ (1 mg mL^−1^) and **(AG)**_**5**_ (1 mg mL^−1^) with **GFRGGRC‐Fluor** (5%, w/w) in sodium phosphate buffer (pH 7, 0.1 M) and PEG‐8 000 (10%, w/v), and (b) after the same sample has been reduced in situ with DTT (20 mM) and reoxidized with H_2_O_2_ (30 mM). Images represent the droplets before bleaching, the frame directly after bleaching, and when recovered. Averages obtained from bleaching three different droplets in the sample. Scale bars = 5 µm. Plots show the relative fluorescence intensity inside the bleached region (black dots) and the data’s fit (red line) of the representative droplet.

The viscosity of the droplets as formed from the *preformed* and well‐characterized dimer **(GFRGGRC)**_**2**_, and after being reduced to dissolve and reoxidized in situ was determined by fluorescence recovery after photobleaching (FRAP) experiments (Figure 4). Before being reduced, the half‐life of fluorescence recovery was 15.3 ± 1.8 s, and after being dissolved and reformed, the half‐life was 12.0 ± 1.8 s. These recovery times are relatively fast, which is further evidence to the fact that the droplets here are in fact liquid in character. Within uncertainty, these two half‐lives are not statistically significantly different (*p* = 0.09), and hence, the viscosity of the droplets before and after reforming is approximately the same (Table S2). Therefore, it is likely that the droplets formed by oxidizing **GFRGGRC** in situ with hydrogen peroxide are of similar character to the ones formed with the *preformed* **(GFRGGRC)**_**2**_.

### 2.3 Selectivity of Peptide Phase Separation to RNA

The purpose of the RNA in the system was to act as a polyanion to interact with the positively charged peptide and undergo LLPS. However, are features of the RNA besides its charge contributing to its ability to undergo LLPS? Previous research by the group determined that a short cationic peptide similar to the one used in this investigation was selective for purine rich decameric RNA **(AG)**_**5**_ over pyrimidine‐rich **(CU)**_**5**_, likely due to differences in the aromaticity of nucleobases [18]. The same discrimination was observed for **(GFRGGRC)**_**2**_, suggesting that the same nucleobase preference applies in the slightly larger peptide analog (Figure S25). The commercially available RNA poly adenylic acid (**poly(A)**) and poly uridylic (**poly(U)**) acids were also tested, both of which formed condensates with the peptide (Figures S25 and S26). However, neither formed as readily as decameric **(AG)**_**5**_, and there was a preference for the **poly(A)** over **(U)**, which again follows the trend for preferential phase separation of cationic peptides with purine over pyrimidine‐rich RNAs [23]. Another commercially available polyanion shown to undergo LLPS with polycations is poly acrylic acid (**PAA**) [24, 25]. It was shown to undergo LLPS with **(GFRGGRC)**_**2**_ at higher peptide concentrations (5 mg mL^−1^). Perhaps more interestingly, discrimination between the RNA and DNA analogs of the decamers was observed. At the same concentration as the RNA, the DNA decamers **(AG)**_**5**,_ **(CT)**_**5**_, **A**_**10**_, and **T**_**10**_ did not form condensates under identical conditions (Figure S25). Therefore, the extra hydroxyl present in the sugar backbone of RNA oligomers must contribute to LLPS with cationic peptides. This extra hydroxyl makes RNA more hydrophilic when compared to DNA, as well as displaying conformational differences in the backbone structure and base stacking [26, 27]. While there is limited research into the difference between RNA and DNA in peptide/nucleic acid condensates, it has been shown previously that RNA interacts more strongly than DNA with arginine‐rich peptides both in the case of forming discrete solution‐phase complexes [28] and LLPS [29]. In the case of the latter, computational modeling was performed, which showed that the RNA displayed a less compacted form in the condensed phase and had the nucleobases more available for hydrogen bonding and *π*–*π* interactions with arginine residues [29, 30].

## 3 Conclusions

In this investigation, a redox‐sensitive condensate system which was able to be reversibly modulated between a one‐ and two‐phase system was demonstrated. This system is derived of “natural” RNA and peptide oligomers, which provides further evidence that the cysteine residues found to flank some naturally occurring disordered regions in proteins could be responsible for modulating the state of membraneless organelles in the cell [31].

The RGG‐repeat‐based peptide, **GFRGGRC**, would not form condensates with the RNA **(AG)**_**5**_, but when oxidized into **(GFRRGC)**_**2**_, either under gentle conditions (*preformed*) or rapidly with hydrogen peroxide in situ, would undergo LLPS into condensates. These condensates could then be dissolved, returning the solution to a single phase, by the addition of reducing agent DTT. These oxidation and reduction cycles could be repeated multiple times, demonstrating a reversible system. The condensates formed from the preformed cystine dimer or after being reduced and oxidized also demonstrated similar viscosity profiles based on FRAP analysis. The control peptide, **GFRGGRS**, did not form condensates upon the addition of oxidizing agent and was hence redox insensitive, highlighting that it is in fact the cysteine residue which acts a redox “handle” in this case.

In addition, the importance of RNA in this system was shown as the oxidized peptide did not form condensates with the DNA analogs of the RNA oligonucleotides and that the oxidized peptide prefers purine over pyrimidine‐based RNA oligomers. This further supports the building evidence that RNA contains important motifs for peptide interactions besides the phosphate backbone and hence its importance in the formation of cellular biomolecular condensates.

Therefore, the first case of a redox‐sensitive condensate requiring RNA is demonstrated. Previous examples either contain no RNA [15, 16] or become redox‐insensitive upon the incorporation of RNA [10]. The model system here therefore serves as a platform for investing further how redox activity in condensate forming proteins originates and can be modulated. Furthermore, the system has potential in the use as a drug delivery vehicle, in particular in the delivery of therapeutics RNAs (e.g., mRNAs and siRNAs) to the cytosol. It has been shown that the reducing environment of the cytosol can be exploited to deliver cargo with a redox‐responsive vehicle [32].

## 4 Experimental Methods

### 4.1 Materials

Solid‐phase peptide synthesis reagents were purchased from Chem‐Impex and Sigma‐Aldrich and used without further purification. Acetonitrile purchased for oligonucleotide synthesis was purchased extremely dry from Sigma‐Aldrich. Solvents for HPLC and LCMS were purchased from Honeywell Burdwick and RCI Labscan and used without further purification. Deuterium oxide used to dissolve NMR samples was purchased from Cambridge Isotope Lab without additional purification. Water is Milli‐Q (resistivity > 18 MΩ) unless otherwise stated.

RNA oligonucleotides **(AG)**_**5**_ and **(CU)**_**5**_ were synthesized as described in previous literature by the primary author [18]. **Poly(A)** and **poly(U)** were purchased from Sigma‐Aldrich as their potassium salts without further purification. DNA oligonucleotides were manufactured by Integrated DNA Technologies and used without further purification.

### 4.2 Peptide Synthesis

The peptide **GFRGGRC** was synthesized according to standard manual solid‐phase techniques. 2‐Chlorotrityl chloride resin (0.405 g, 1.14 mmol g^−1^, 0.462 mmol) was added to a polypropylene syringe fitted with a filter and swelled in dichloromethane (15 min). Fmoc‐Cys(Trt)‐OH (0.811 g, 1.39 mmol, 3 eq.) and *N*,*N*‐diisopropylethylamine (0.64 mL, 3.70 mmol, 8 eq.) were dissolved in dichloromethane (2 mL) and *N*,*N*‐dimethylformamide (2 mL). This solution was drawn into the syringe and shaken overnight (r.t.). The resin was washed with *N*,*N*‐dimethylformamide (3 × 1 min), piperidine in *N*,*N*‐dimethylformamide (20%, v/v, 1 × 5 min, 1 × 10 min), and *N*,*N*‐dimethylformamide (5 × 1 min). Fmoc‐Arg(Pbf)‐OH (0.899 g, 1.39 mmol, 3 eq.) was dissolved in 1‐hydroxybenzotriazole hydrate and *N*,*N*,*N*′,*N′*‐tetramethyl‐*O*‐(1*H*‐benzotriazol‐1‐yl)uronium hexafluorophosphate, *O*‐(benzotriazol‐1‐yl)‐*N*,*N*,*N′*,*N′*‐tetramethyluronium hexafluorophosphate (0.5 M, 2.77 mL, 1.39 mmol, 3 eq.), and *N*,*N*‐diisopropylethlamine (0.48 mL, 2.77 mmol, 6 eq.) and drawn into the syringe and shaken (r.t. 45 min). The coupling was confirmed by the Kaiser test, where a small amount of resin was combined with a few drops of each reagent: (A) ninhydrin (0.5 g) in ethanol (10 mL), (B) phenol (8 g) in ethanol (2 mL), and (C) aqueous potassium cyanide (0.01 M, 0.2 mL) in pyridine (9.8 mL). The five remaining amino acids, Fmoc‐Gly‐OH (0.412 g, 3 equiv.), Fmoc‐Gly‐OH (0.412g, 3 equiv.), Fmoc‐Arg(Pbf)‐OH (0.899 g, 3 equiv.), Fmoc‐Phe‐OH (0.536 g, 3 equiv.) and Fmoc‐Gly‐OH (0.424 g, 3 equiv.) were each dissolved in *N*,*N*‐diisopropylethylamine (0.48 mL, 6 equiv.) and a coupling solution of 1‐hydroxybenzotriazole hydrate and *N*,*N*,*N′*,*N′*‐tetramethyl‐*O*‐(1*H*‐benzotriazol‐1‐yl)uronium hexafluorophosphate in *N*,*N*‐dimethylformamide (0.5 M, 2.8 mL, 3 equiv.). For each amino acid coupling, the amino acid solution was drawn into the syringe to be shaken (45 min), followed by the Kaiser test to determine the success of coupling. Each successful coupling was followed by washes with *N*,*N*‐dimethylformamide (3 × 1 min), 20% piperidine in *N*,*N*‐dimethylformamide solution (20%, v/v, 1 × 5 min, 1 × 10 min), and *N*,*N*‐dimethylformamide (5 × 1 min). Upon completion of the final Fmoc deprotection, the resin was washed in dichloromethane (3 × 1 min) and methanol (3 × 1 min) and briefly dried under nitrogen. The peptide was cleaved from the resin using a cleavage solution of trifluoroacetic acid (7.2 mL, 90%), thioanisole (0.4 mL, 5%), ethane‐1,2‐dithiol (0.24 mL, 3%), and anisole (0.16 mL, 2%) and shaken (3 h). Upon completion, the contents of the syringe were ejected into ice‐cold diethyl ether (~20 mL), and the resulting precipitate was collected by centrifugation (4000 rpm, 5 min). The supernatant was discarded, and the crude product was washed with ice‐cold diethyl ether and centrifuged twice more. The precipitate was dissolved in Milli‐Q water and lyophilized to give the crude product as a fluffy yellow solid. The dry, crude peptide was dissolved in Milli‐Q water (14.4 mL, 95%) and acetonitrile (1.6 mL, 5%) and purified by reversed‐phase HPLC on a C‐18 column. The mobile phase varied in composition from 95% solvent A: Milli‐Q water with formic acid (0.1%, v/v) and 5% solvent B: acetonitrile with formic acid (0.1%, v/v) to 95% solvent B over 35 min. The product eluted at 8 min, and the fractions were collected, combined, and lyophilized to give the pure **GFRGGRC** (72 mg, 0.096 mmol, 21%). HRMS (ESI^+^) m/z: [M+H]^+^ calculated for C_30_H_50_N_13_O_8_S: 752.3621 found: 752.3623. ^1^H NMR (600 MHz, D_2_O) *δ* 7.32–7.27 (m, 2H, Phe‐Ar‐H), 7.27–7.22 (m, 1H, Phe‐Ar‐H), 7.22–7.17 (m, 2H, Phe‐Ar‐H), 4.38–4.28 (m, 2H, *α*‐CH), 4.19 (dd, *J* = 8.6, 5.8 Hz, 1H, *α*‐CH), 3.95–3.77 (m, 4H, Gly‐CH_2_), 3.77–3.64 (m, 2H, Gly‐CH_2_), 3.11 (t, 2H, Arg‐*δ*‐CH_2_), 3.08 (t, 2H, Arg‐*δ*‐CH_2_), 2.99 (d, 2H, Phe‐*β* *β*‐CH_2_), 2.83 (t, 2H, Cys‐*β*‐CH_2_), 1.86–1.65 (m, 4H, Arg‐*β*‐CH_2_), 1.65−1.58 (m, 2H, Arg‐*γ*‐CH_2_), 1.50–1.38 (m, 2H, Arg‐*γ*‐CH_2_).

The peptide **GFRGGRS** was synthesized according to standard automated solid‐phase synthesis techniques with a Liberty Blue Microwave Peptide Synthesizer with HT‐12 attachment. Amino acid solutions were prepared at 0.2 M in the following total volumes: Fmoc‐Arg(Pbf)‐OH (26 mL), Fmoc‐Gly‐OH (19 mL), and Fmoc‐Phe‐OH (7 mL). *N*,*N′*‐diisopropylcarbodiimide (activator, 25 mL) and ethyl 2‐cyano‐2‐(hydroxyimino)acetate (activator base, 25 mL) were prepared at 1 M each. These solutions were loaded onto the synthesizer at the appropriate positions, and wash solvent (*N*,*N*‐dimethylformamide) and deprotection solvent (piperidine in *N*,*N*‐dimethylformamide, 20%, v/v) were filled to the appropriate level. Fmoc‐Ser(tBu)‐wang resin (0.472 g, 0.51 mmol g^−1^, 0.1 mmol) was loaded onto the HT‐12 attachment, suspended in dichloromethane and *N*,*N*‐dimethylformamide (1:1, ~10 mL). The synthesis was then run at the default 0.1 mmol scale settings, with double coupled arginines and final deprotection settings.

Once the synthesis was complete, the resin was transferred to a 12 mL polypropylene syringe fitted with a filter. The resin was washed with dichloromethane (3 × 6 mL) and methanol (3 × 6 mL) before being dried under a stream of nitrogen. A cleavage cocktail consisting of trifluoroacetic acid (5.7 mL, 95%), triisopropylsilane (0.15 mL, 2.5%), and water (0.15 mL, 2.5%) was drawn into the syringe and shaken (3 h, r.t.). Upon completion, the contents of the syringe were ejected into ice‐cold diethyl ether (~20 mL), and the resulting precipitate was collected by centrifugation (4000 rpm, 5 min). The supernatant was discarded, and the crude product was washed with ice‐cold diethyl ether and centrifuged twice more. The precipitate was dissolved in Milli‐Q water and lyophilized to give the crude product as a fluffy off‐white solid. The dry, crude peptide was dissolved in Milli‐Q water (95%) and acetonitrile (5%) at a concentration of 5 mg mL^−1^ and purified by reversed‐phase HPLC on a C‐18 column. The mobile phase varied in composition from 95% solvent A: Milli‐Q water with formic acid (0.1%, v/v) and 5% solvent B: acetonitrile with formic acid (0.1%, v/v) to 95% solvent B over 35 min. The product eluted at 11 min, and the fractions were collected, combined, and lyophilized to give the pure **GFRGGRS** (21 mg, 0.028 mmol, 28%). HRMS (ESI^+^) m/z: C_30_H_50_N_13_O_9_ [M+H]^+^ calculated: 736.3849, found: 736.3838. ^1^H NMR (600 MHz, D_2_O) *δ* 7.32–7.18 (m, 5H, Phe‐Ar‐H), 4.23–4.15 (m, 1H, *α*‐CH), 4.00–3.79 (m, 4H, Gly‐CH_2_), 3.76 (d, *J* = 2.3 Hz, 2H, Gly‐CH_2_), 3.75–3.72 (m, 2H, Ser‐*β*‐CH_2_), 3.16–3.05 (m, 4H, Arg‐*δ*‐CH_2_), 3.00 (d, *J* = 8.0 Hz, 2H, Phe‐*β*‐CH_2_), 1.90–1.70 (m, 2H, Arg‐*β*‐CH_2_), 1.70–1.62 (m, 2H, Arg‐*β*‐CH_2_), 1.58 (m, 2H, Arg‐*γ*‐CH_2_), 1.45 (m, 2H, Arg‐*γ*‐CH_2_).

**GFRGGRC** (10 mg) was dissolved in ammonium carbonate buffer (0.1 m, pH 8) at a concentration of 0.1 mg mL^−1^ (100 mL). The solution was stirred, with air bubbled through solution. The progression of the reaction was monitored by reversed‐phase HPLC, and took 8 days to reach completion. The solution was then lyophilized, and once dry was redissolved in water and acetonitrile (95:5, 4 mL). The sample was then purified by reversed‐phase HPLC on a C‐18 column. The mobile phase varied in composition from 95% solvent A: Milli‐Q water with formic acid (0.1%, v/v) and 5% solvent B: acetonitrile with formic acid (0.1%, v/v) to 95% solvent B over 35 min. The product eluted at 9 min, and the fractions were collected, combined, and lyophilized to give the pure **(GFRGGRC)**_**2**_ (7.5 mg, 0.005 mmol, 75%). C_60_H_97_N_26_O_16_S_2_: 1501.7012 found: 1501.7047 ^1^H NMR (600 MHz, D_2_O) *δ* 7.30–7.11 (m, 10H, Phe‐Ar‐H), 4.59–4.48 (m, 2H, *α*‐CH), 4.39–4.31 (m, 2H, *α*‐CH), 4.20–4.13 (m, 2H, *α*‐CH), 3.93–3.75 (m, 8H, Gly‐CH_2_), 3.75–3.69 (m, 2H, Gly‐CH_2_), 3.54–3.33 (m, 10H, Gly/Arg‐CH_2_), 3.16–3.08 (m, 2H, Arg‐*δ*‐CH_2_), 3.08−2.99 (m, 8H Cys/Phe‐CH_2_), 2.99–2.88 (m, 4H, Arg‐CH_2_), 1.84–1.75 (m, 4H, Arg‐CH_2_), 1.67–1.60 (m, 2H, Arg‐CH_2_), 1.60–1.46 (m, 6H, Arg‐CH_2_), 1.46–1.37 (m, 4H, Arg‐CH_2_).

**GFRGGRC** (2.0 mg, 0.0027 mmol, 1 eq.) and tris(2‐carboxyethyl)phosphine (0.3 mg, 0.0013 mmol, 0.5 eq.) were dissolved in sodium phosphate buffer (0.1 M, pH 6.8, 0.8 mL) and stirred (2 h, r.t.). Fluorescein‐5,6‐maleimide (3.41 mg, 3 eq.) was dissolved in *N*,*N*‐dimethylformamide (0.2 mL) and added to the first solution. The solution was left to stir in the dark (18 h, r.t) and once complete was filtered and directly purified by reversed‐phase HPLC. The product eluted at 16 min, the fractions were collected and lyophilized to afford **GFRGGRC‐Fluor** as a bright orange solid (2.2 mg, 69%). HRMS (ESI^+^) m/z: C_54_H_64_N_14_O_15_S [M + 2H]^2+^ calculated: 590.2193, found: 590.2196. ^1^H NMR (600 MHz, D_2_O) *δ* 8.35 (s, 2H), 7.88 (d, *J* = 7.5 Hz, 1H), 7.84 (s, 1H), 7.76 (d, *J* = 2.4 Hz, 1H), 7.06–6.98 (m, 6H), 6.88 (d, *J* = 8.5 Hz, 2H), 6.82 (d, *J* = 7.7 Hz, 2H), 6.63 (s, 1H), 6.58 (s, 1H), 6.52 (d, *J* = 7.5 Hz, 2H), 6.51 (s, 1H), 4.69 (s, 1H), 4.36 (t, *J* = 3.7 Hz, 2H), 4.27 (dd, *J* = 9.3, 5.0 Hz, 2H), 4.19–4.10 (m, 3H), 4.02 (t, *J* = 4.5 Hz, 2H), 3.85–3.78 (m, 8H), 3.77–3.70 (m, 3H), 3.70–3.58 (m, 6H), 3.49–3.43 (m, 2H), 3.29 (dd, *J* = 14.3, 4.2 Hz, 2H), 3.12 (d, *J* = 8.3 Hz, 2H), 3.03 (td, *J* = 6.9, 1.4 Hz, 5H), 2.96 (q, *J* = 8.0 Hz, 6H), 2.92 (s, 4H), 2.90–2.85 (m, 2H), 2.84–2.79 (m, 2H), 2.77 (d, *J* = 0.9 Hz, 3H), 1.86 (s, 2H), 1.79 (ddd, *J* = 13.8, 10.1, 5.3 Hz, 3H), 1.76–1.67 (m, 4H), 1.67–1.42 (m, 13H), 1.39 (s, 2H), 1.17–1.11 (m, 2H).

### 4.3 Plate Reader Turbidity Measurements

Stock solutions of peptide and RNA were prepared at 12 mg mL^−1^ in sodium phosphate buffer (0.1 M, pH 7). Stock solutions of PEG‐8000 are prepared at 40% (w/v) in sodium phosphate buffer (0.1 M, pH 7). The pH of concentrated stock solutions was confirmed to remain at pH = 7. Stock solutions of DTT, *tris*‐(2‍‐‍carboxyethyl)phosphine hydrochloric acid salt, and hydrogen peroxide (as diluted from 30%, v/v solution) were each prepared at 1 M concentration in sodium phosphate buffer (0.1 M, pH 7). However, the pHs were not adjusted after dilution into the buffer, and hence, the final pHs of these stock solutions of reducing and oxidizing agent are likely not pH 7. These stock solutions were pipetted in triplicate in a 384‐well polystyrene plate to make the final concentrations of desired species at a total volume of 60 µL. A ThermoFisher VarioSkan Lux Plate reader was used to measure the absorbance of each well at 450 and 610 nm at 25 °C.

### 4.4 Brightfield Microscopy

A Nikon TE2000‐U inverted microscope with a Plan Apo Vc 100× oil immersion lens (NA 1.40) with a CoolLED pT‐100 LED source recorded with a pco.edge 4.2 sCMOS camera was used for all brightfield microscopy. The RNA and peptide samples were prepared at 12 mg mL^−1^ in sodium phosphate buffer (0.1 M, pH 7). The (poly)ethylene glycol (PEG) samples were prepared at 40% (w/v) in sodium phosphate buffer (0.1 M, pH 7). The pH of concentrated stock solutions was confirmed to remain at pH = 7. Each peptide/RNA/PEG ratio was made up to 60 µL and added to a glass slide. In order to oxidize and reduce the sample, concentrated (1 M) solutions of DTT and hydrogen peroxide (H_2_O_2_) were prepared and added to make the desired final concentrations of these species.

### 4.5 Confocal Microscopy

A Zeiss LSM 880 confocal microscope with a 100× oil immersion objective (NA 1.4) with Argon ion 488 nm laser was used for confocal imaging. The RNA and peptide samples were prepared at 12 mg mL^−1^ in sodium phosphate buffer (0.1 M, pH 7). For the peptide stock solution, 5% (w/w) of **GFRGGRC‐Fluor** with respect to **GFRGGRC** or **GFRGGRS** was added. The (poly)ethylene glycol (PEG) samples were prepared at 40% (w/v) in sodium phosphate buffer (0.1 M, pH 7). The pH of concentrated stock solutions was confirmed to remain at pH = 7. Each peptide/RNA/PEG ratio was made up to 60 µL and added to a glass slide. In order to oxidize and reduce the sample, concentrated (1 M) solutions of DTT and hydrogen peroxide (H_2_O_2_) were prepared and added to make the desired final concentrations of these species.

### 4.6 FRAP

The sample preparation was the same as that for confocal microscopy. The region of interest was bleached using the 405 and 488 nm laser (100% power), and 200 frames were recorded immediately after using the 488 nm laser. In each sample, three different condensates were analyzed. For each sample, three regions of interest were selected: sample (bleached) and two unbleached areas, one of a condensate which was not bleached as a control and one of the background.

The ZEN FRAP analysis module was used to fit the data and determine the fluorescence recovery values, similar to what was done in our previous work [18]. The software fit the data to the following equation: *I* = *I*_0_(1−e^−*t*/*τ*^) where, *I* = fluorescence intensity, *I*_0_ = initial fluorescence intensity, *t* = time, and *τ* = the fluorescence recovery time constant. The halftime of the recovery was calculated from *t*_1/2_ = *τ*ln(2).

## Funding

This study was supported by Australian Research Council (DP220101847, DP250100081).

## Conflicts of Interest

The authors declare no conflicts of interest.

## Supporting information

Supplementary Material

## Data Availability

The data that support the findings of this study are available from the corresponding author upon reasonable request.
