# Supplementary material for: Reversible Redox‐Responsive Peptide Condensates With RNA
Source: Chembiochem. 2026 Aug 21;27(16):e70513. doi: 10.1002/cbic.70513 (PMC13495114; doi:10.1002/cbic.70513)
Supplement: Supplementary file 1 [file CBIC-27-e70513-s001.pdf]

Supplementary Information for:

Reversible redox-responsive peptide condensates with RNA

Anika L. Moller[a] and Pall Thordarson*[a]

[a] Dr. A.L. Moller and Prof. P. Thordarson
School of Chemistry and the RNA Institute
University of New South Wales
Sydney, NSW, 2052
p.thordarson@unsw.edu.au

.

Additional Methods for Peptide Purification and Characterisation

Preparative High Performance Liquid Chromatography

For peptide purification, reverse phase HPLC was performed with a Shimadzu Prominence UFLC HPLC system equipped with a 20AB pump and an SPD-20A Photo diode array detector. A C-18 Interstil ODS-4 5 μm 20 x 150 mm preparative column was used, and the mobile phase consisted of A: Milli-Q™ water with 0.1% formic acid v/v and B: acetonitrile 0.1% with formic acid (v/v). The solvent flow rate was 5 mL min^{-1}, and a gradient program was used where the concentration of solvent B varied from 5-95% over 30 min. The chromatogram was viewed at 254 nm for peptides and 490 nm for fluorescein conjugated peptides.

Liquid Chromatography Mass Spectrometry (LCMS)

A Shimadzu LCMS2020 system was used, with a Shimadzu Shim-pack Scepter C18-120 5 μm 4.6 x 150 mm column. The mobile phase consisted of A: Milli-Q™ water with 0.1% formic acid (v/v) and B: acetonitrile 0.1% with formic acid (v/v). The solvent flow rate was 0.8 mL min^{-1} with a gradient program was used where the concentration of solvent B varied from 5-95% over 35 min. The chromatogram was viewed at 254 nm for peptides and 490 nm for fluorescein conjugated peptides. A mass spectrum from 20-2000 m/z was recorded continuously, so the identity of the peptide could be confirmed with low resolution.

Nuclear Magnetic Resonance (NMR) Spectroscopy

Spectra were recorded on a Bruker Avance III Neo (500 MHz), Bruker Avance III (600 MHz), or Bruker Avance III HD (600 MHz); All spectra were recorded at 298 K. Samples were dissolved in the specified solvents with signals referenced to residual solvent peaks. Signals were recorded in the following order where relevant: chemical shift (ppm from residual solvent signal) identified integral, multiplicity, coupling constants (J in Hz) and assignments. All NMR data were acquired using TopSpin and analysed using Topspin or MestReNova . Multiplicities are assigned as singlet (s), doublet (d), triplet (t), quartet (q), pentet (p), sextet (sx), doublet of doublet (dd), doublet of triplet (dt), triplet of doublet (td), multiplet (m).

High Resolution Mass Spectrometry

For peptide samples, the high-resolution mass spectrometry was performed by the Bioanalytical Mass Spectrometry Facility (BMSF) at UNSW. A Orbitrap XL instrument was used in positive and negative ESI modes to obtain high resolution mass spectra.

Analytical High Performance Liquid Chromatography

Analytical HPLC was performed with a Shimadzu LCMS2020 (disconnected from the mass spectrometer due to buffer salts remaining in the sample) instrument with LC30AD pumps, fitted with a Shimadzu Shim-pack Scepter C18-120 5 μm, 4.6 x 150 mm column. The flow rate was 0.5 mL min^{-1}, and the mobile phase consisted of A: water with 0.1%,

v/v formic acid, and B: acetonitrile with 0.1%, v/v formic acid. The concentration of solvent B was slowly raised on a gradient from 5% to 95% over 30 min, before being brought back down to 5% at 35 min.

Characterisation Data

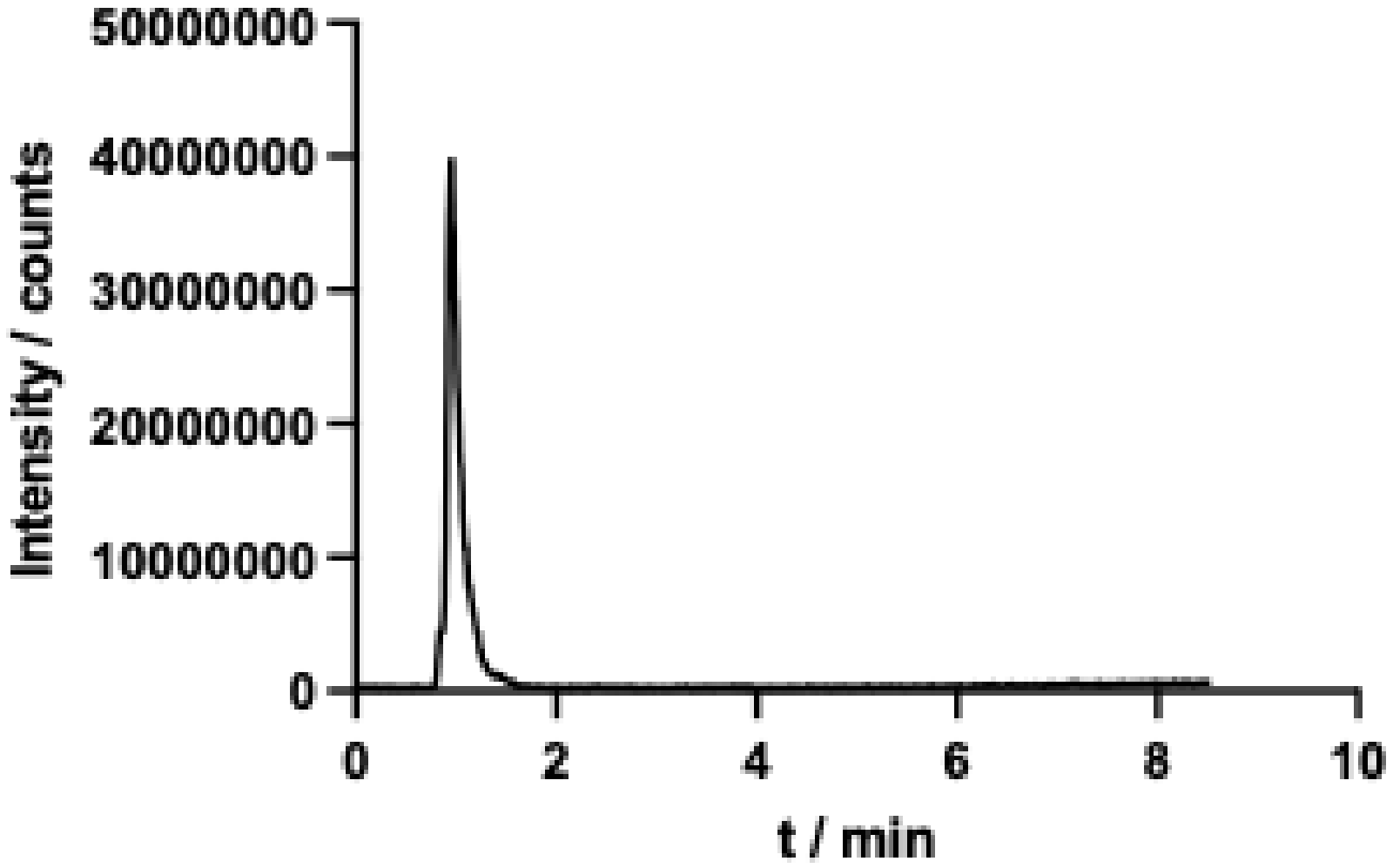

Figure S1. TIC trace of **GFRGGRC**.

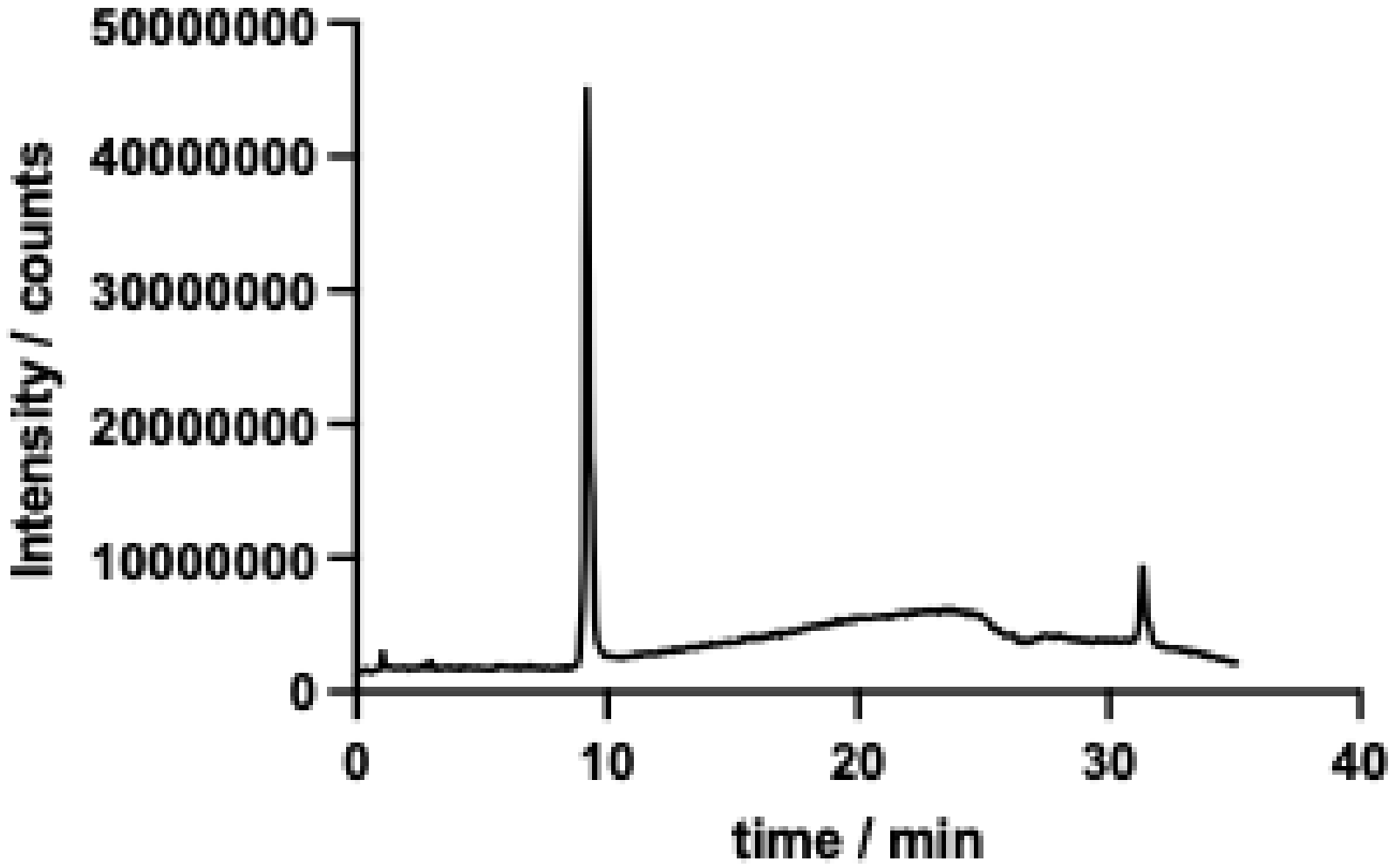

Figure S2. TIC trace of **$(GFRGGRC)_2$**.

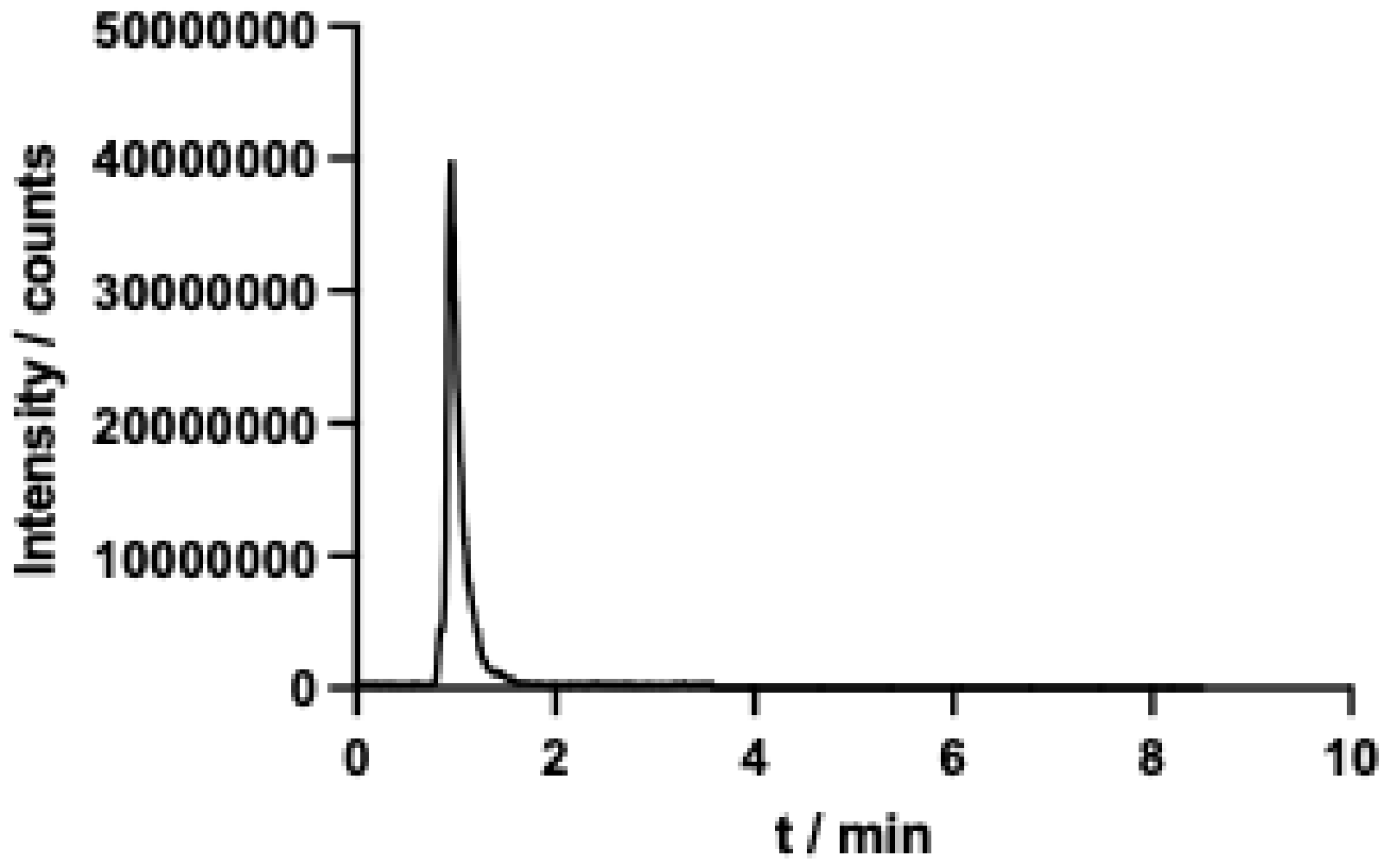

Figure S3. TIC trace of **GFRGGRS**.

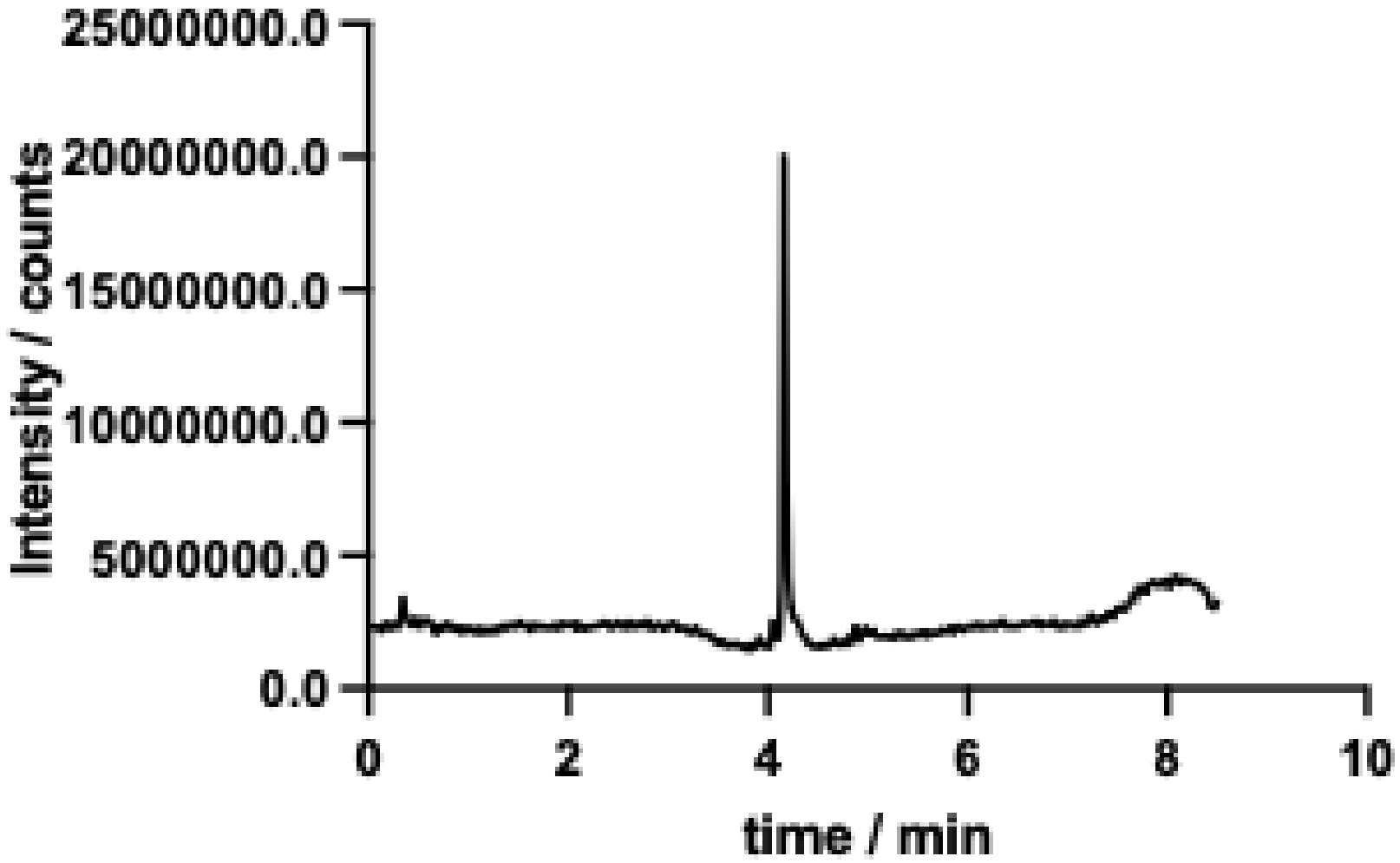

Figure S4. TIC trace of **GFRGGRC-Fluor**.

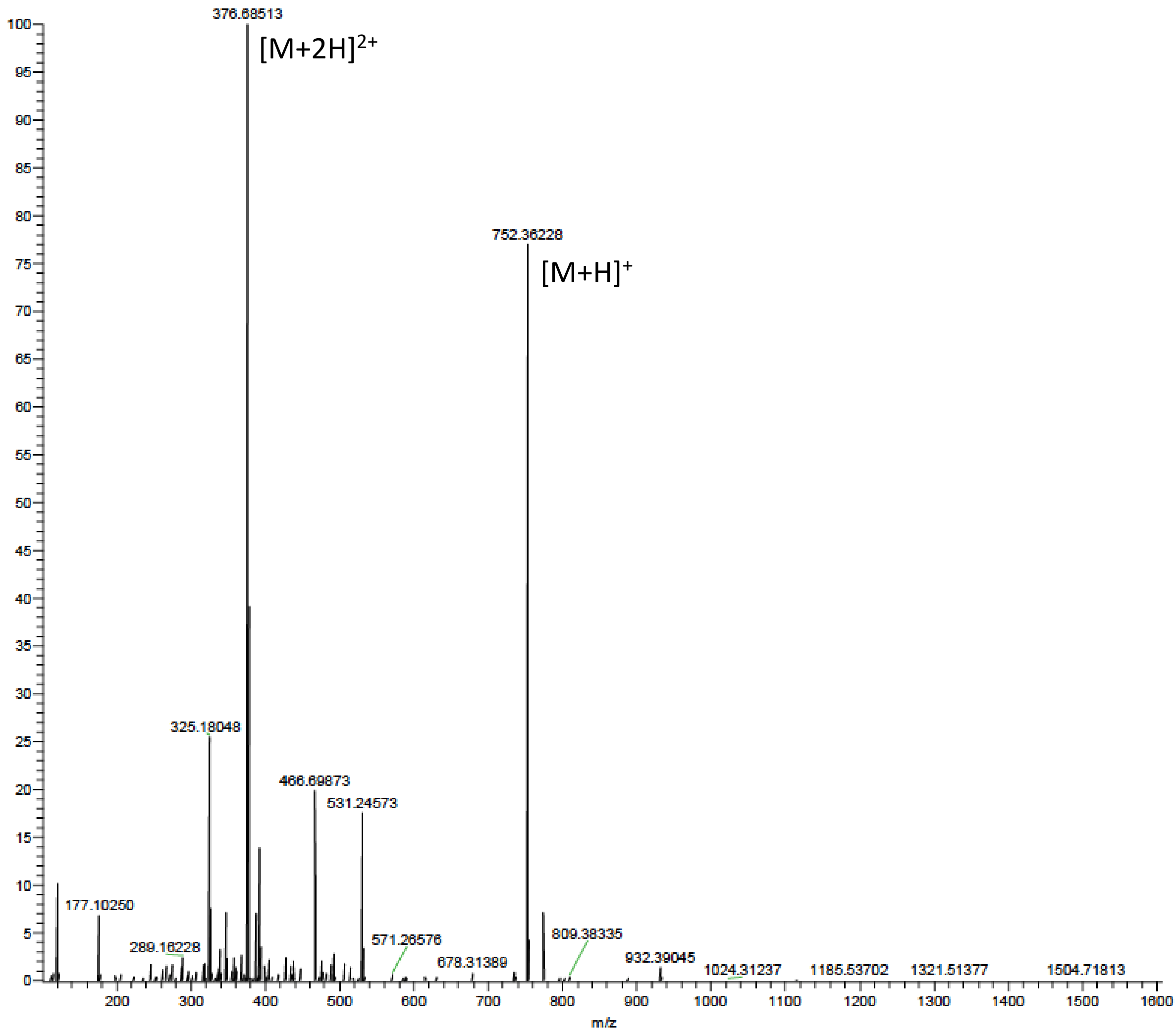

Figure S5. Positive ESI mass spectrum of **GFRGGRC**.

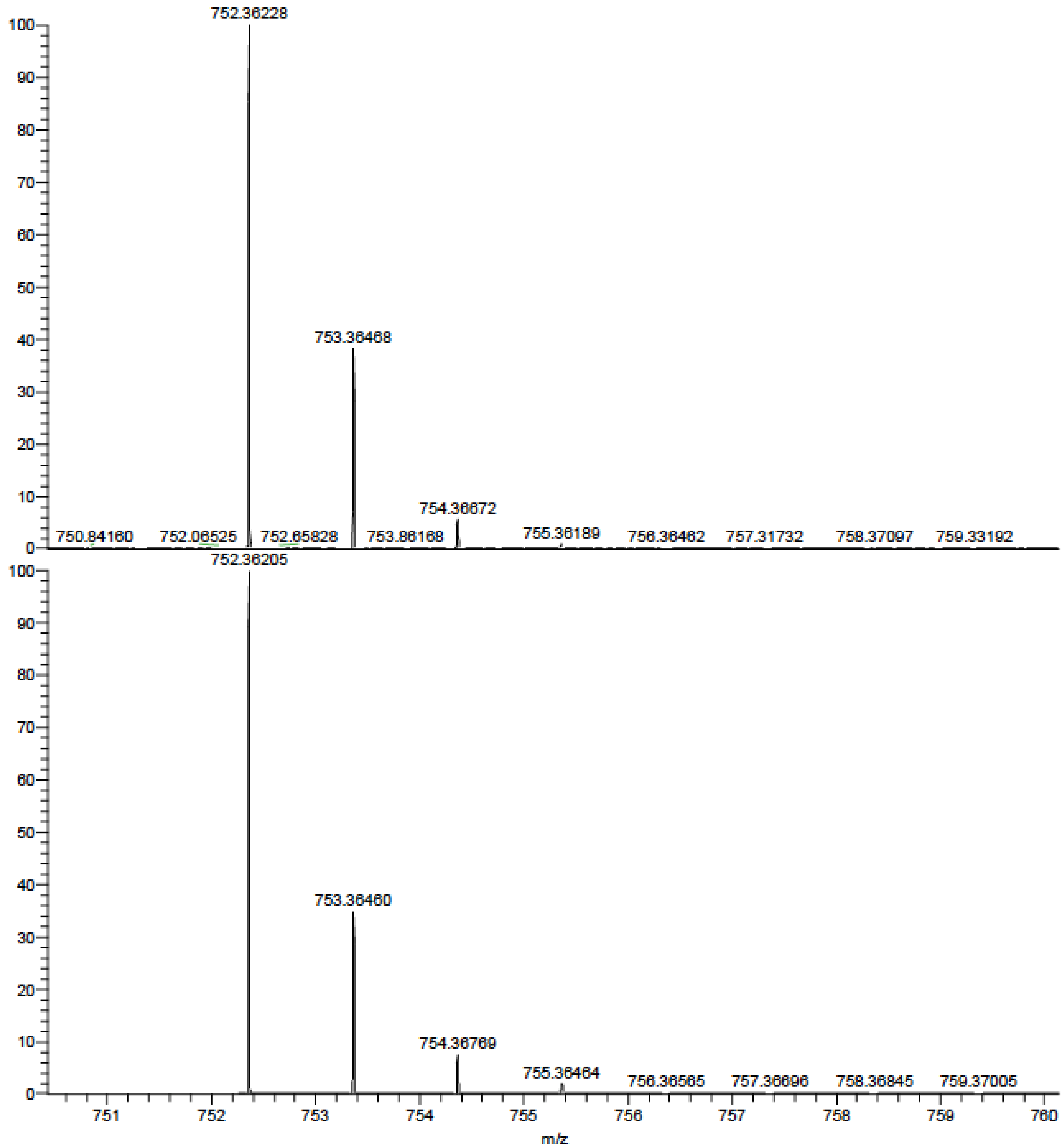

Figure S6. Zoomed spectrum of **GFRGGRC** [M+H]⁺ (top) and simulated spectrum (bottom).

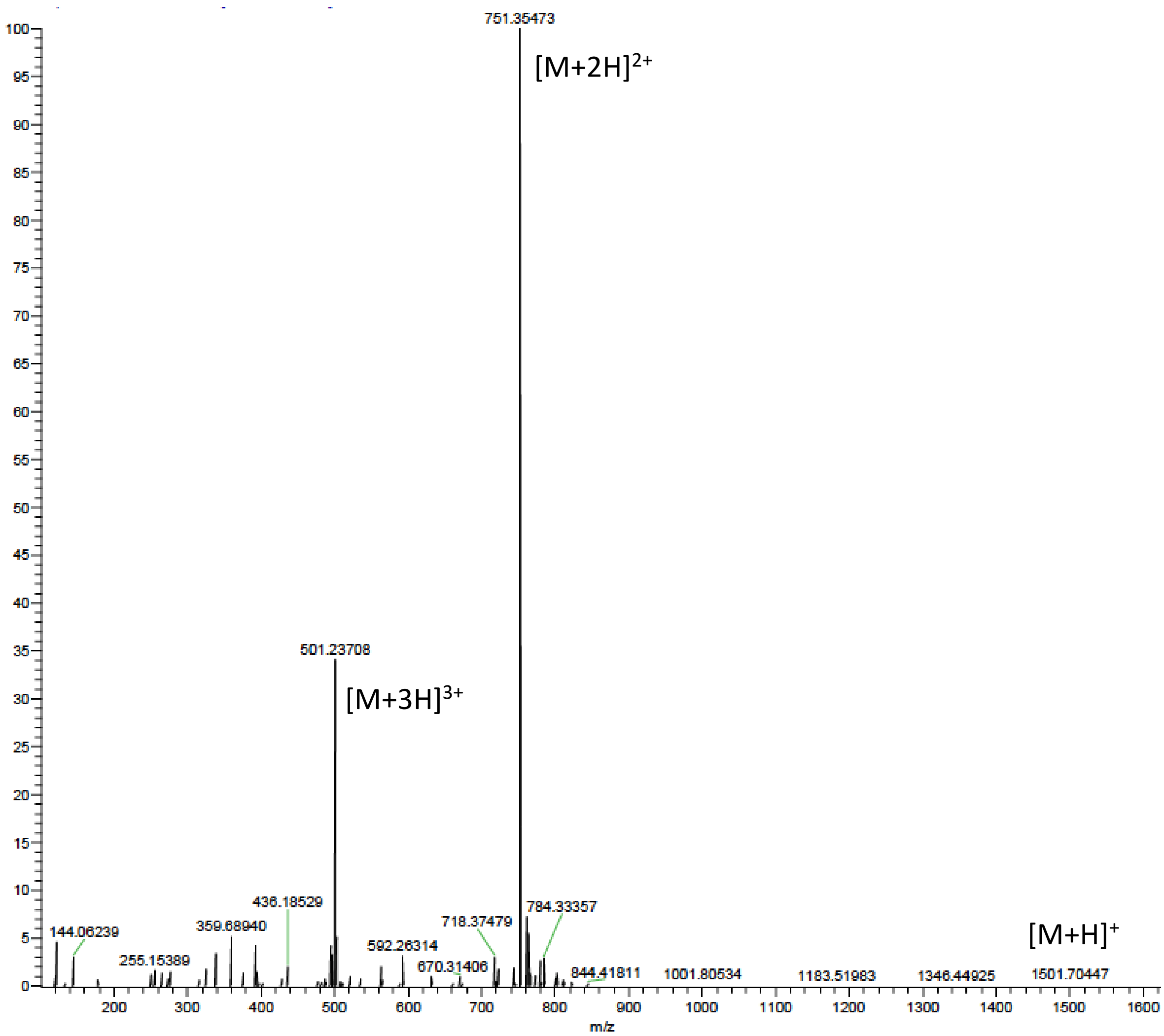

Figure S7. Positive ESI mass spectrum of **(GFRGGRC)$_2$**.

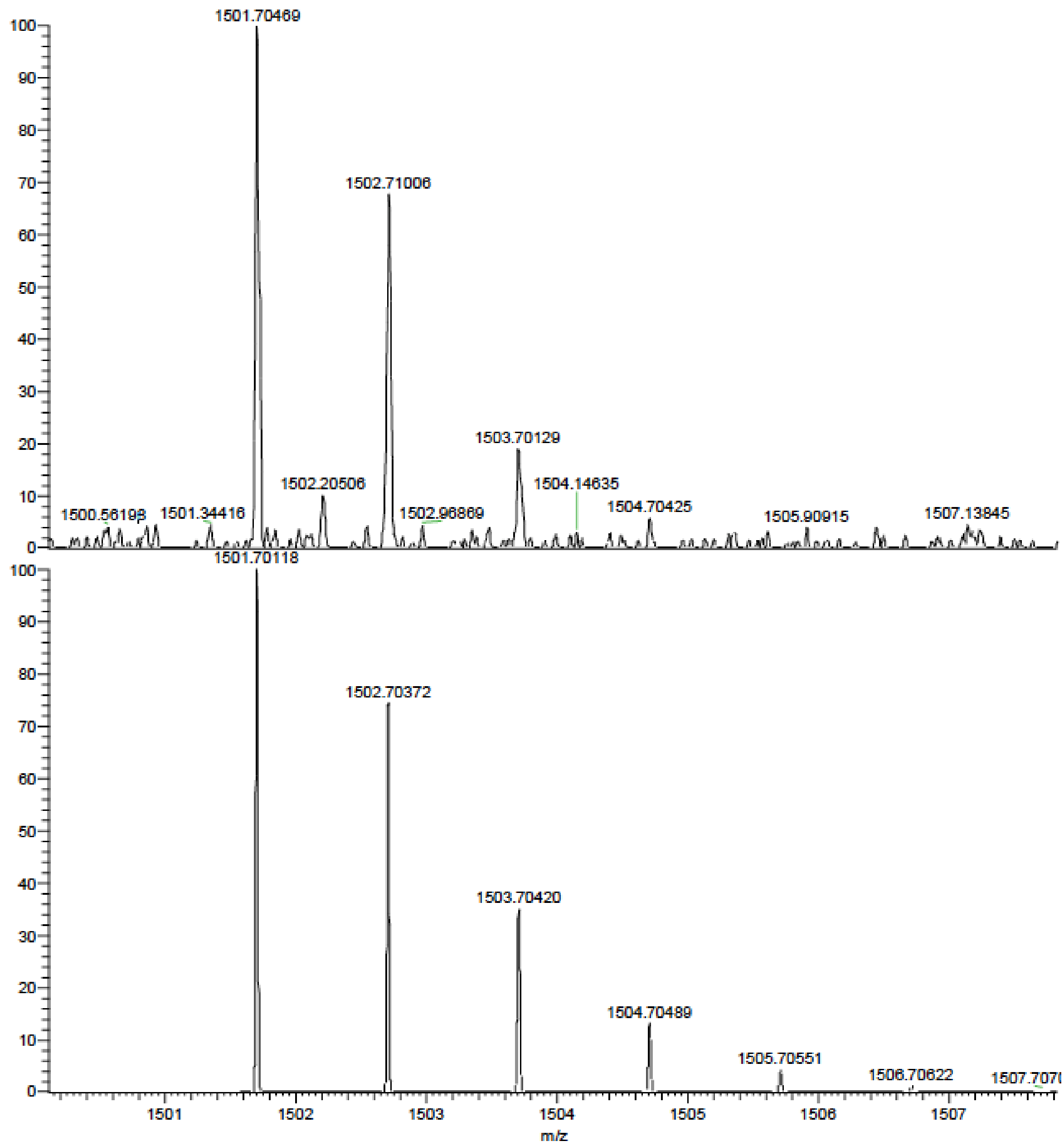

Figure S8. Zoomed spectrum of **(GFRGGRC)₂** [M+H]⁺ (top) and simulated spectrum (bottom).

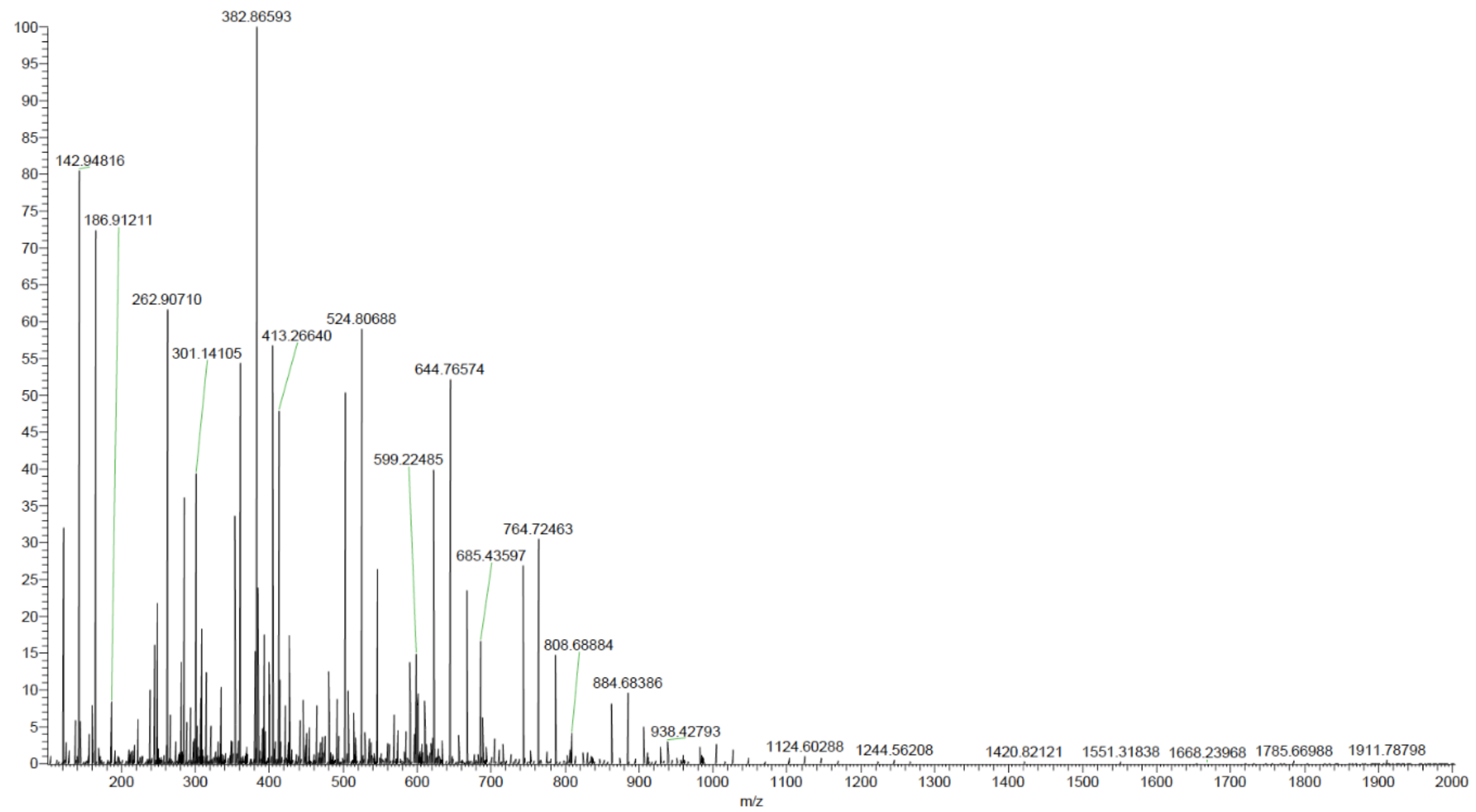

Figure S9. Full ESI mass spectrum of **GFRGGRC-Fluor**. Very low concentration of sample gives many background peaks.

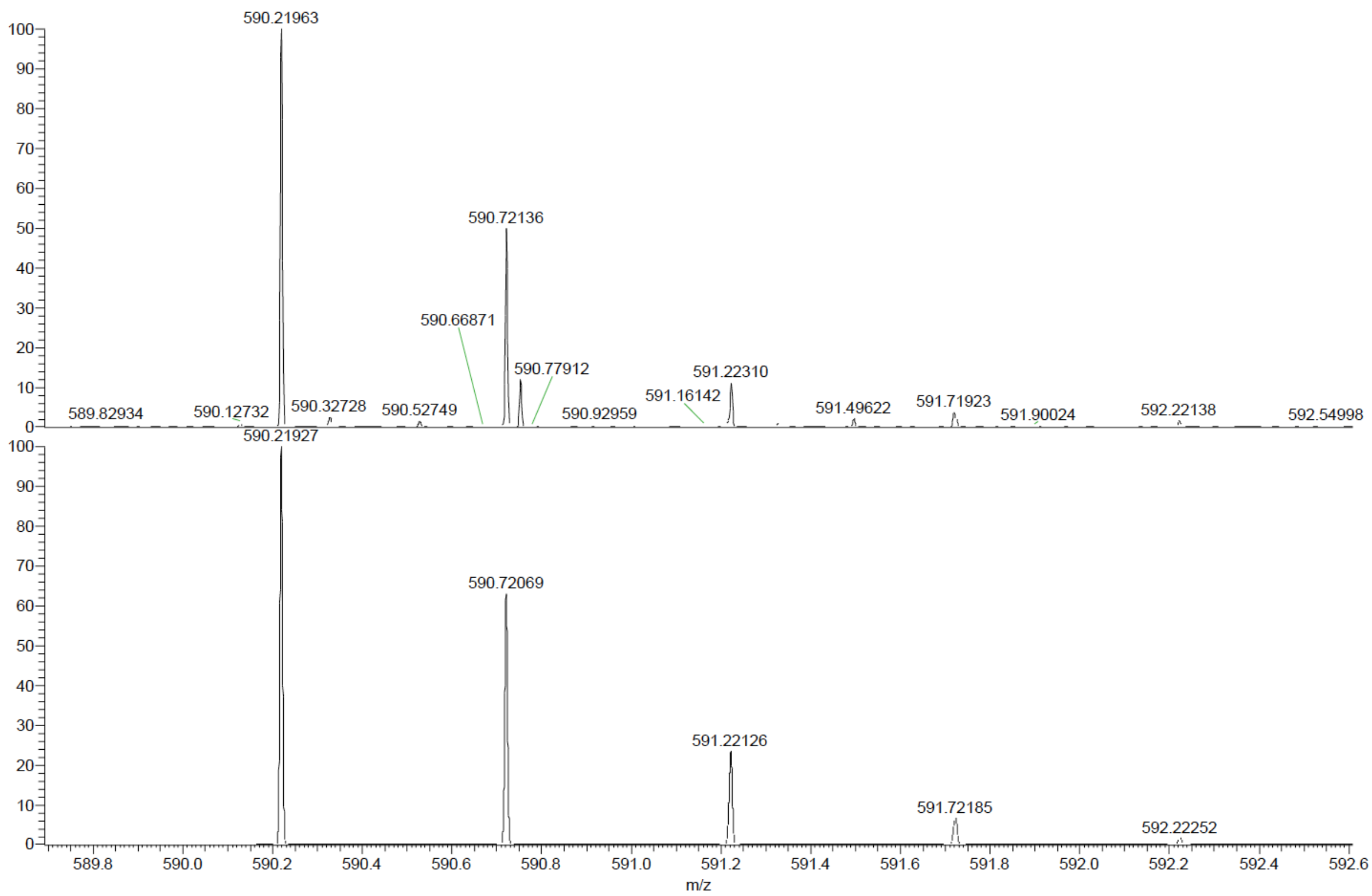

Figure S10. Zoomed Spectrum of **GFRGGRC-Fluor** $[M+2H]^{2+}$ (top) and simulated spectrum (bottom).

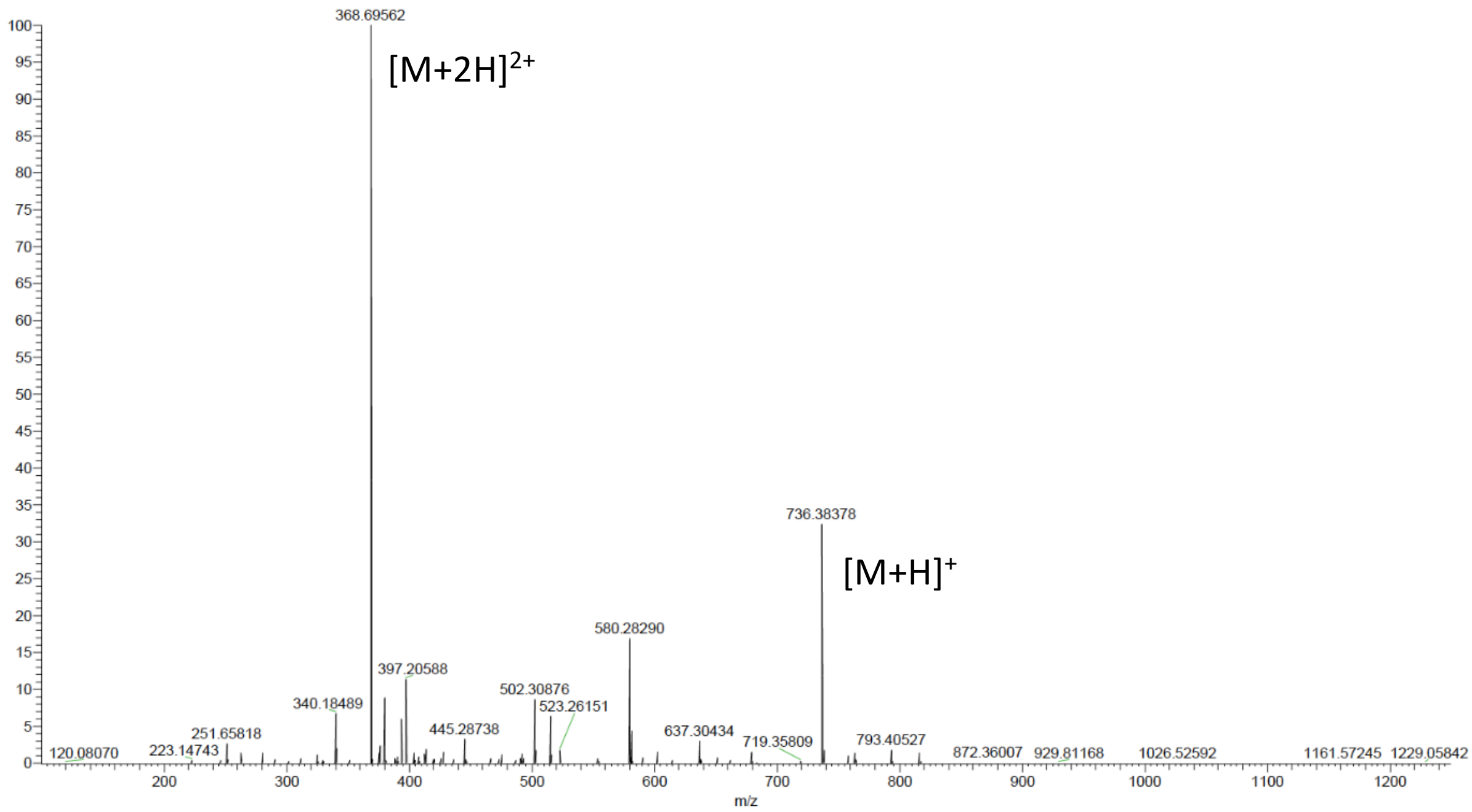

Figure S11. Positive ESI mass spectrum of **GFRGGRS**.

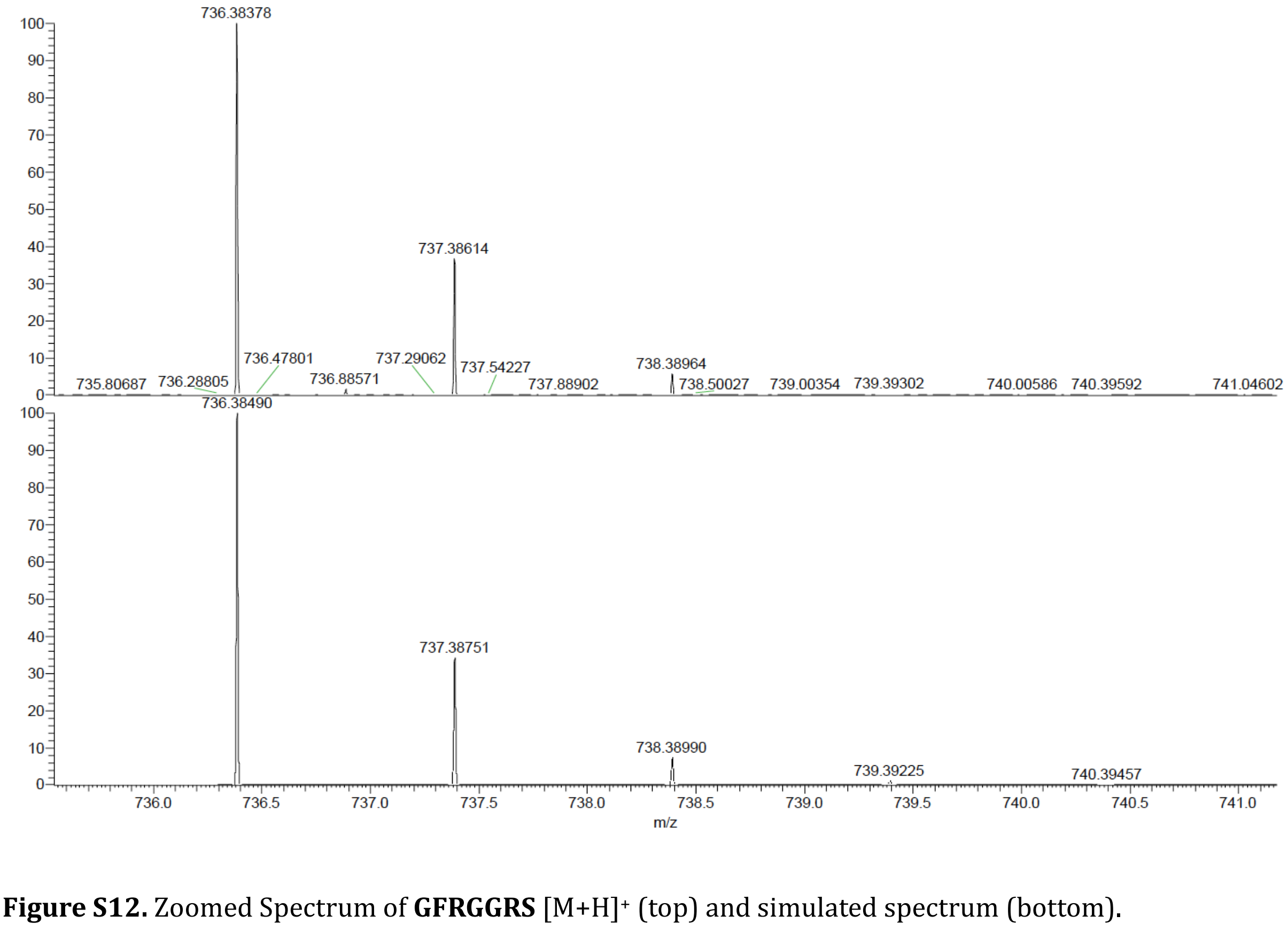

Figure S12. Zoomed Spectrum of **GFRGGRS** [M+H]+ (top) and simulated spectrum (bottom).

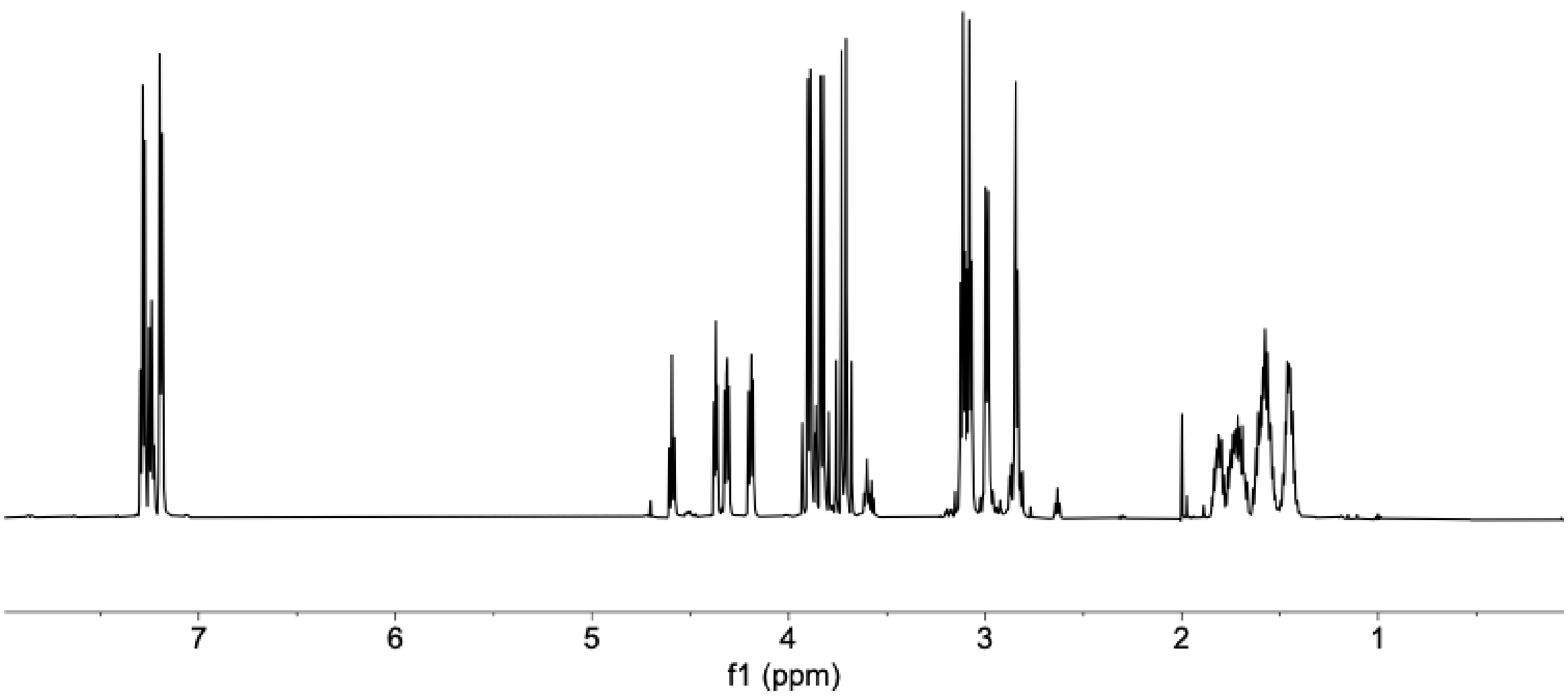

Figure S13. ^{1}H NMR (600 MHz, D_2O) spectra of **GFRGGRC.**

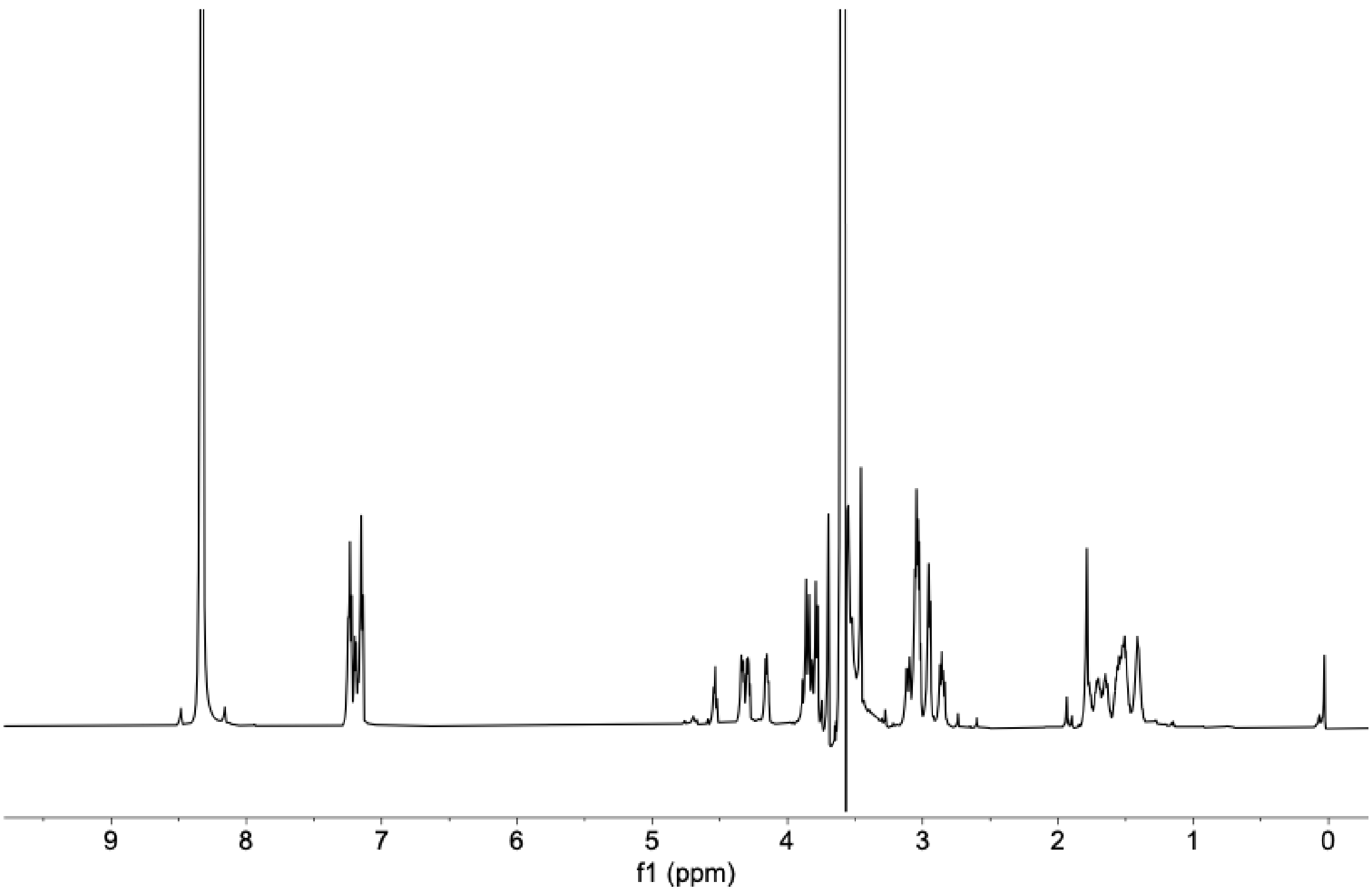

Figure S14. ^{1}H NMR (600 MHz, D_2O) spectra of **(GFRGGRC)$_2$.**

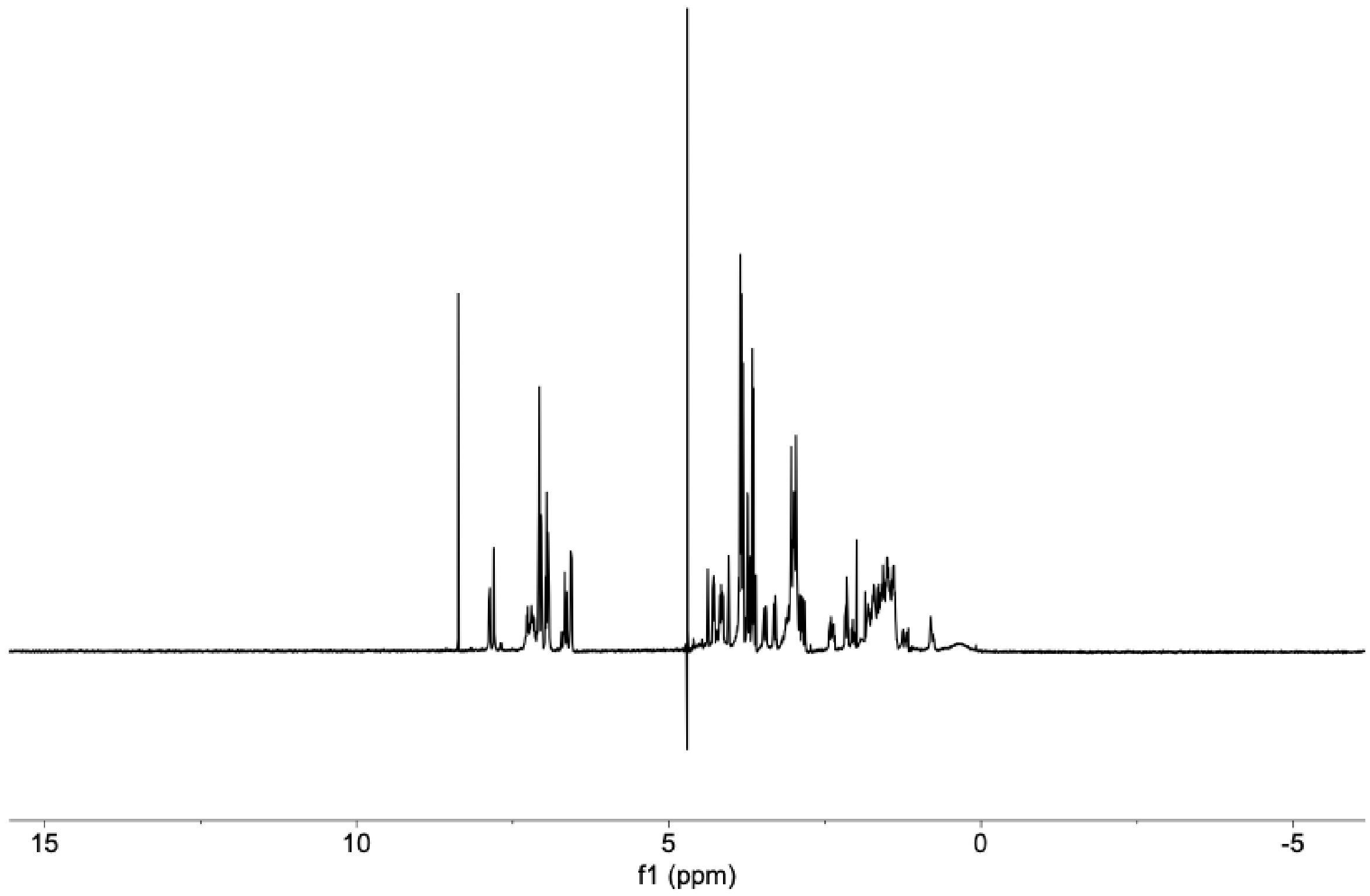

Figure S15. ^{1}H NMR (600 MHz, D2O) spectra of **GFRGGRC-Fluor**.

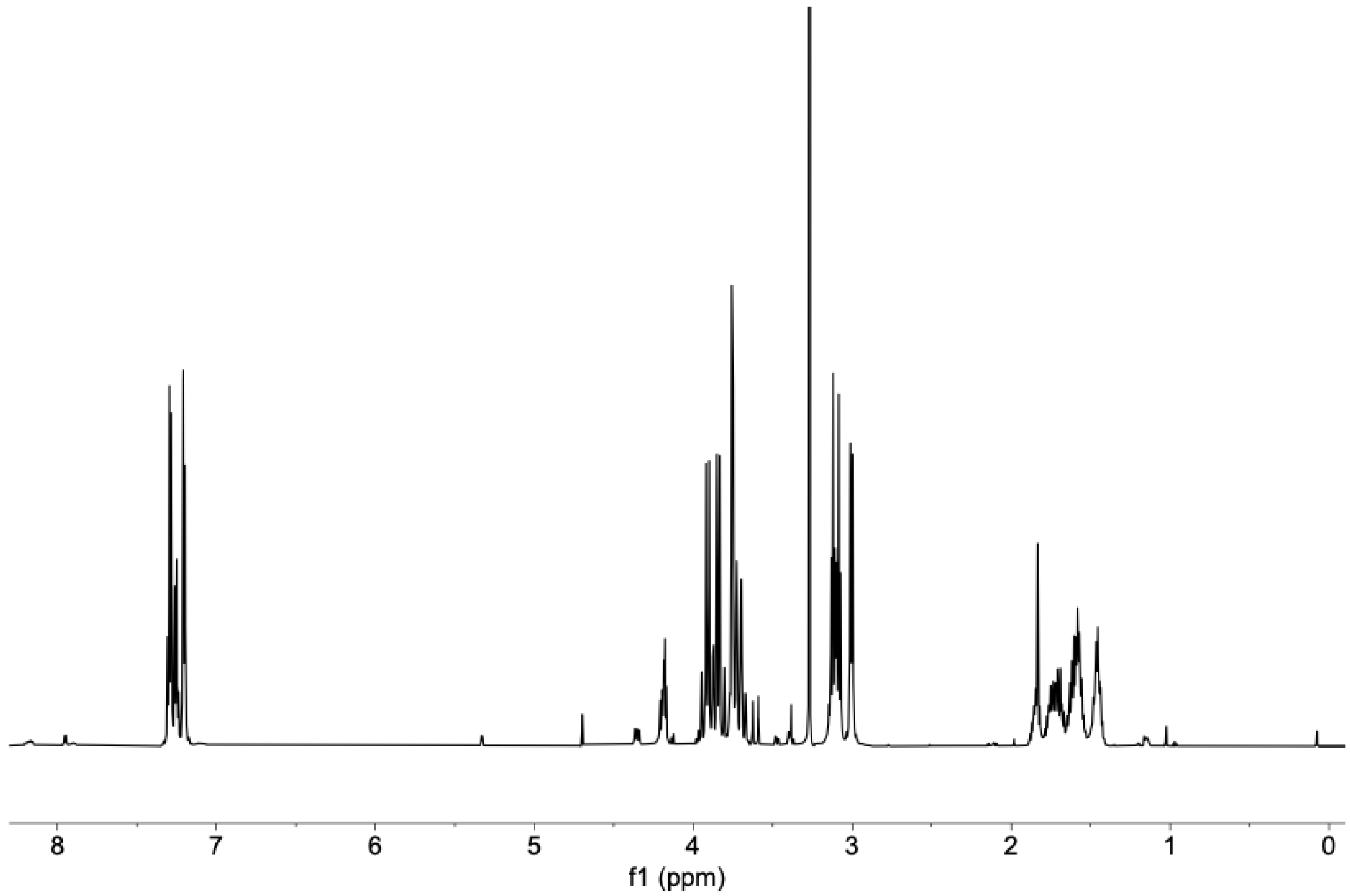

Figure S16. ^{1}H NMR (600 MHz, D_2O) spectra of **GFRGGRS**.

Table S1. Table of buffer type, pH and how long it took for peptide oxidation reaction to reach completion, according to how long the monomer peak took to disappear. For those denoted as 14+, this indicates that after 14 days, the starting material peak was still present.

Buffer[a] (50 mM)	pH	Time until completion / d
Tris	8	4
NaPi	7.5	14+
KPi	7.5	14+
HEPES	8	14+
NH_4CO_3	8	8

[a]Abbreviations of buffer names are as follows: Tris, tris(hydroxymethyl)aminomethane; NaPi, sodium phosphate; KPi, potassium phosphate; HEPES, 4-(2-hydroxyethyl)-1-piperazineethanesulfonic acid; NH_4CO_3, ammonium carbonate.

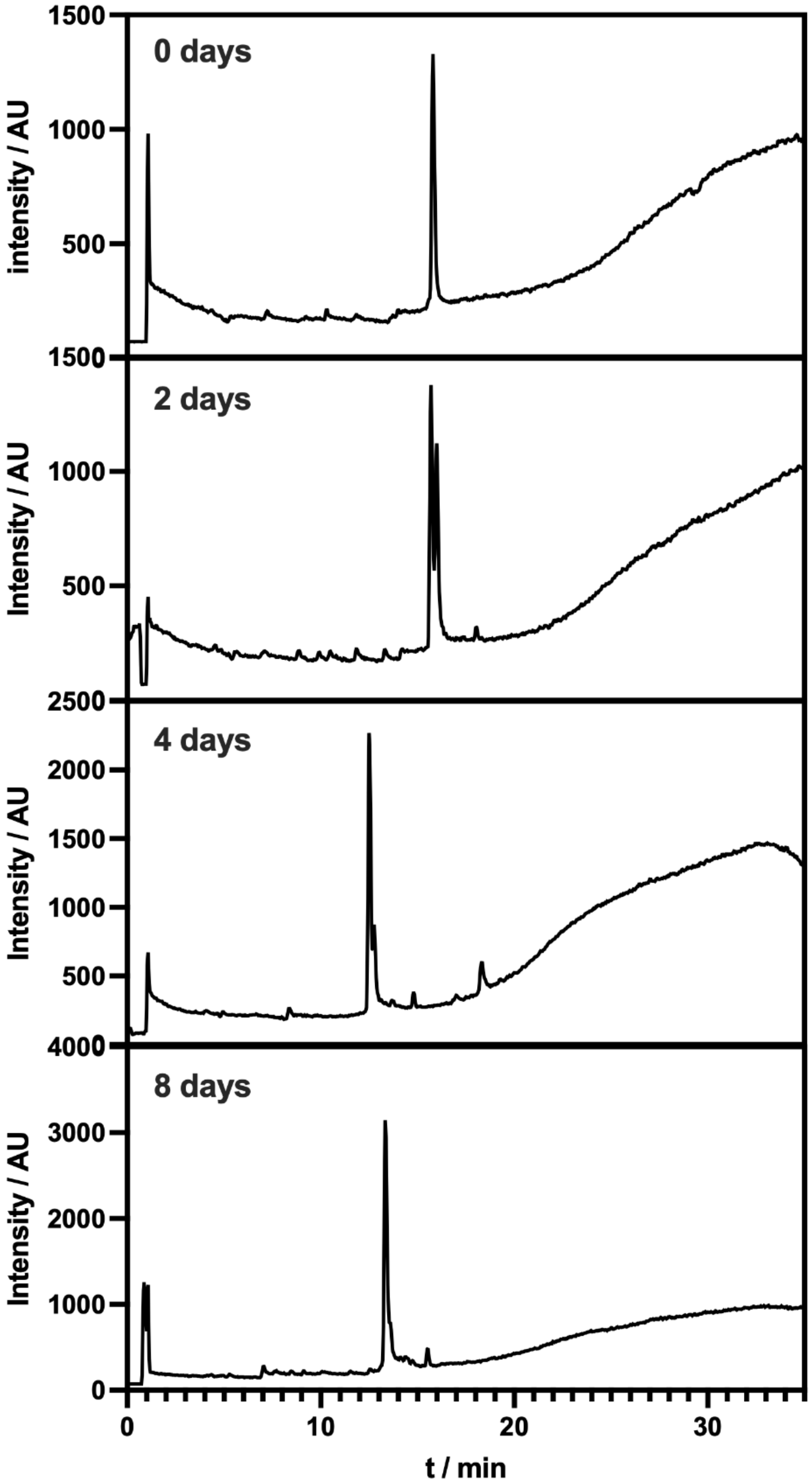

Figure S17. Analytical reversed-phase HPLC chromatogram viewed at 254 nm of **GFRGGRC**'s oxidation into **(GFRGGRC)**$_2$ over 8 days in ammonium carbonate (50 mM, pH 8). The mobile phase consisted of A: water with formic acid (0.1%, v/v) and B: acetonitrile with formic acid (0.1%, v/v), and the concentration of B varied from 5-95%. Note the retention time shifts early, notably after 2 days. This is likely attributable due to the fact this instrument is communal and the mobile phase solvents might not have been made up identically between these runs.

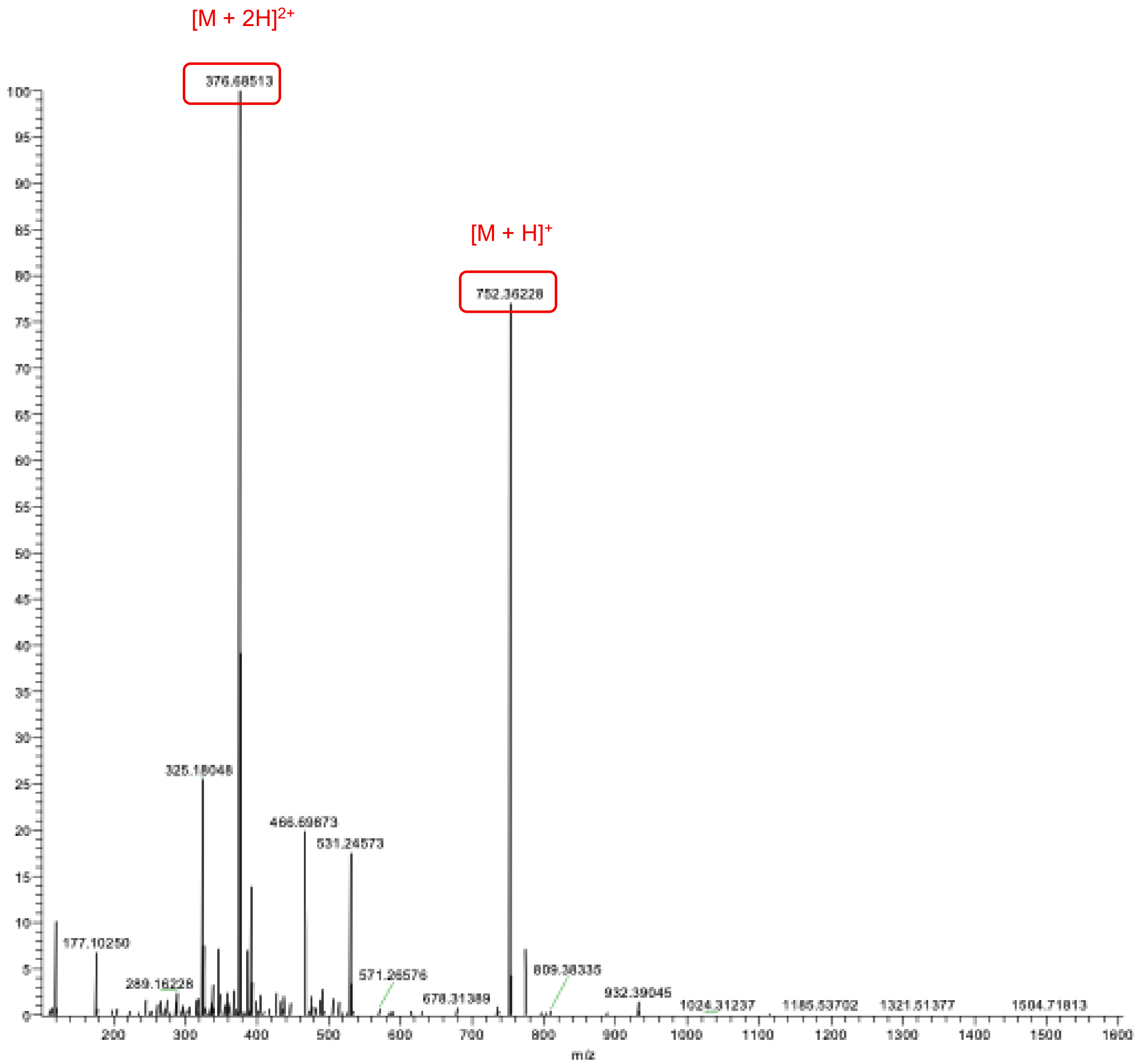

Figure S18. Electrospray ionisation (ESI) positive mode mass spectrum of **GFRGGRC** from 100-2000 m/z.

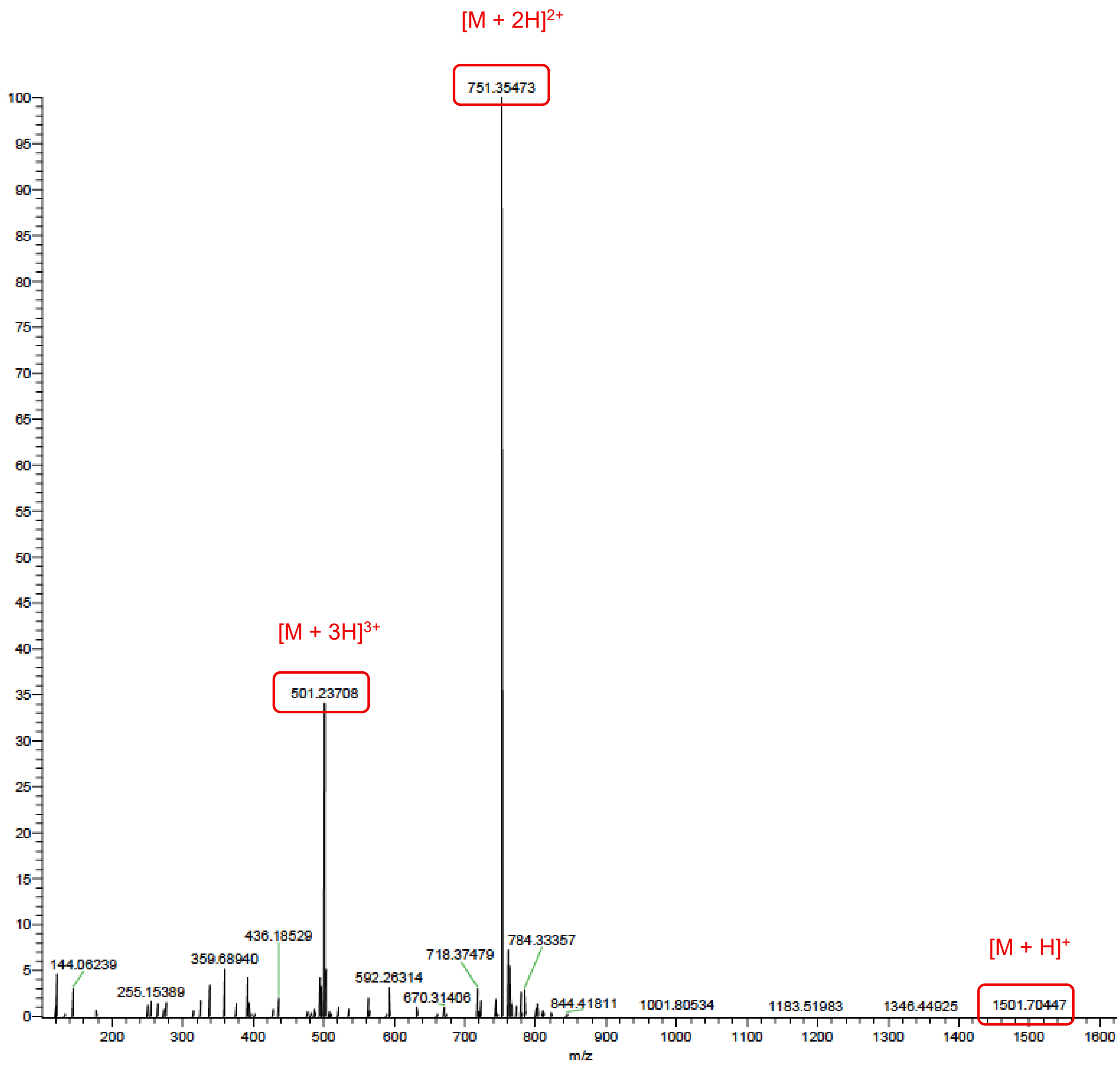

Figure S19. Electrospray ionisation (ESI) positive mode mass spectrum of (**GFRGGRC)**$_2$ from 100-2000 m/z.

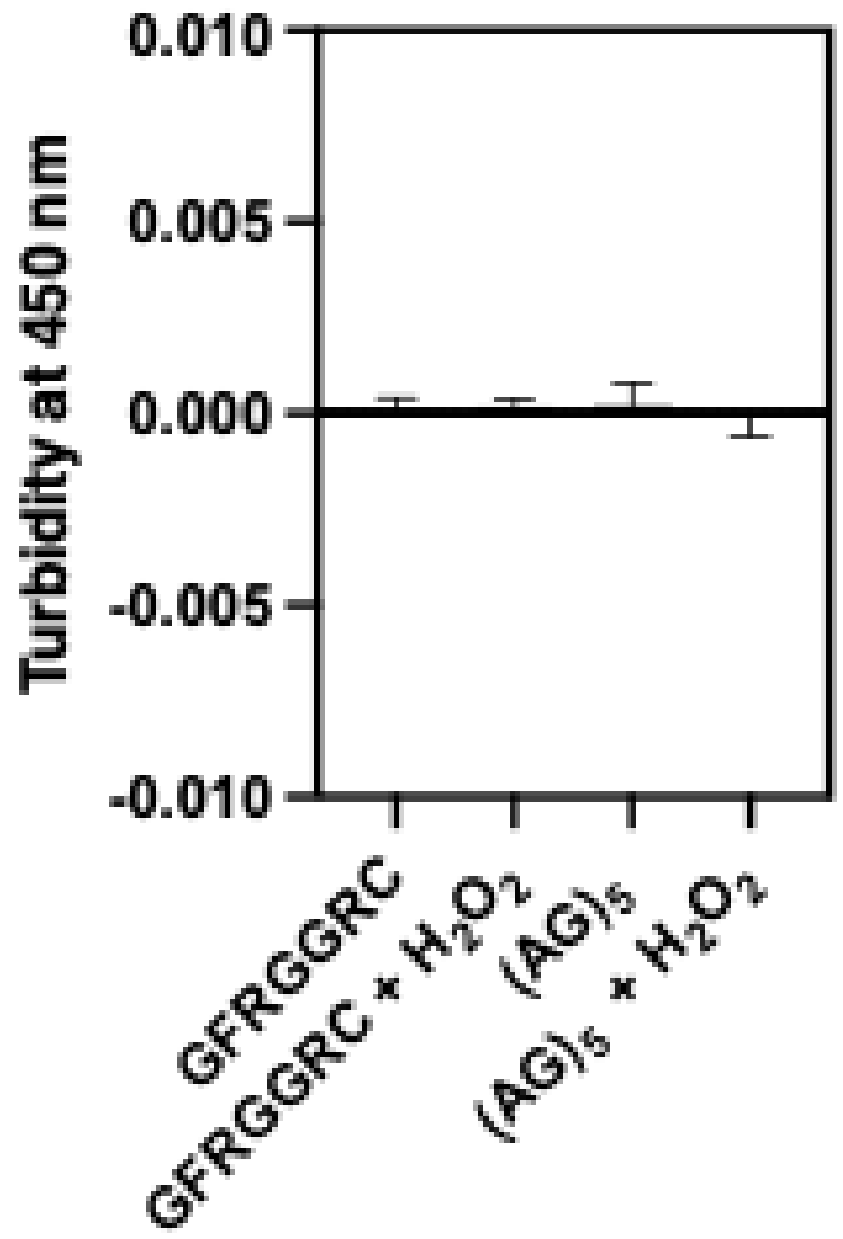

Figure S20. Turbidity measurements of peptide and RNA as prepared and under oxidising conditions.

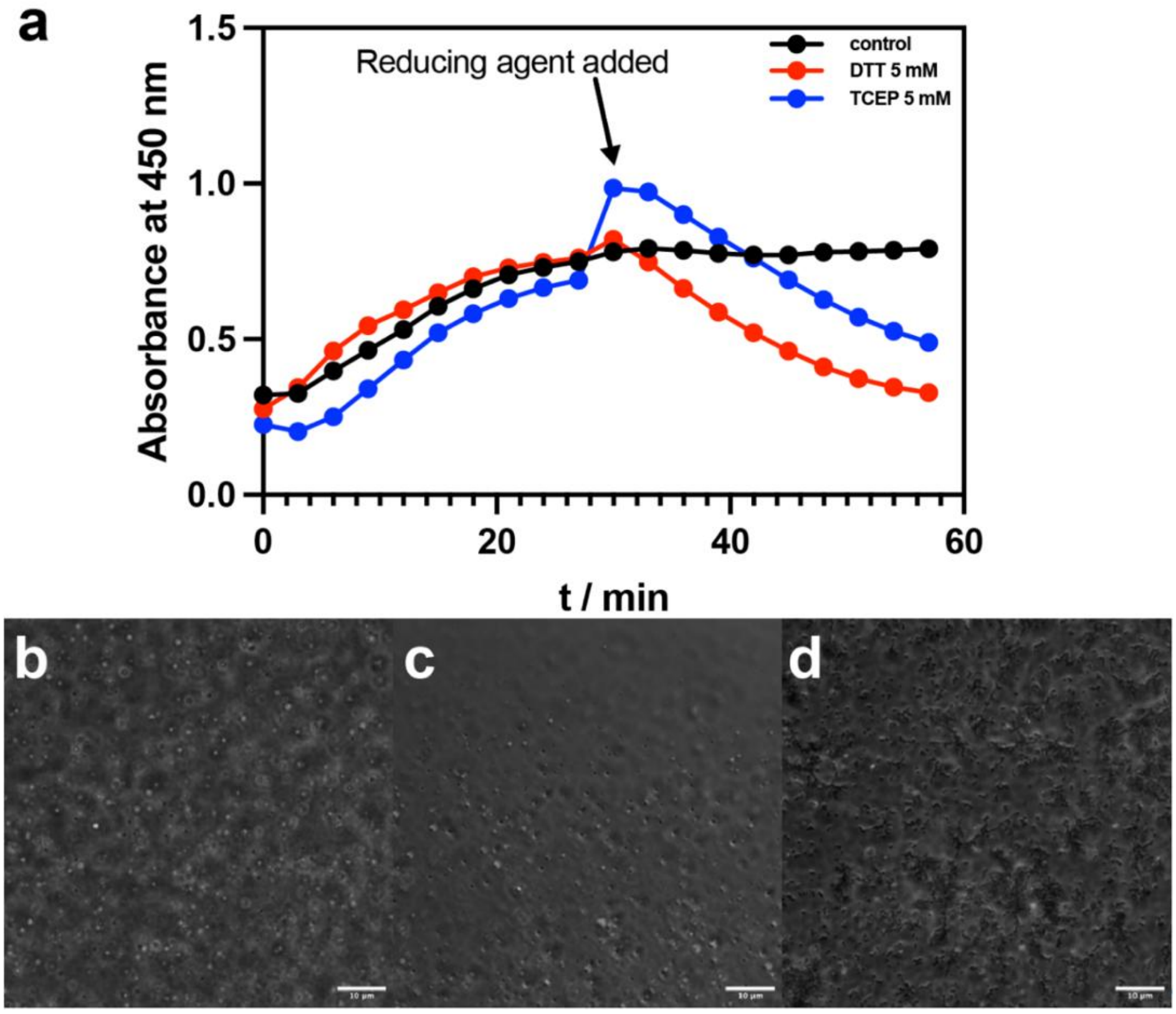

Figure S21. (a) Turbidity over time of condensate samples with **$(AG)_5$** (1 mg mL^{-1}) and **$(GFRGGRC)_2$** (1 mg mL^{-1}) in sodium phosphate buffer (pH 7, 0.1 M) with PEG-8000 (10%, w/v) after the addition of 5 mM H_2O_2 at t = 0. A reducing agent was added at t = 30 min, either nothing (black), D,L-dithiothreitol (5 mM, red), or *tris*-(2-carboxyethyl)phosphine hydrochloric acid (5 mM, blue). Brightfield microscopy images of condensates samples (b) after addition of hydrogen peroxide and 30 min incubation, (c) immediately after adding D,L-dithiothreitol and (d) immediately after adding *tris*-(2-carboxyethyl)phosphine hydrochloric acid. Scale bars = 10 μm.

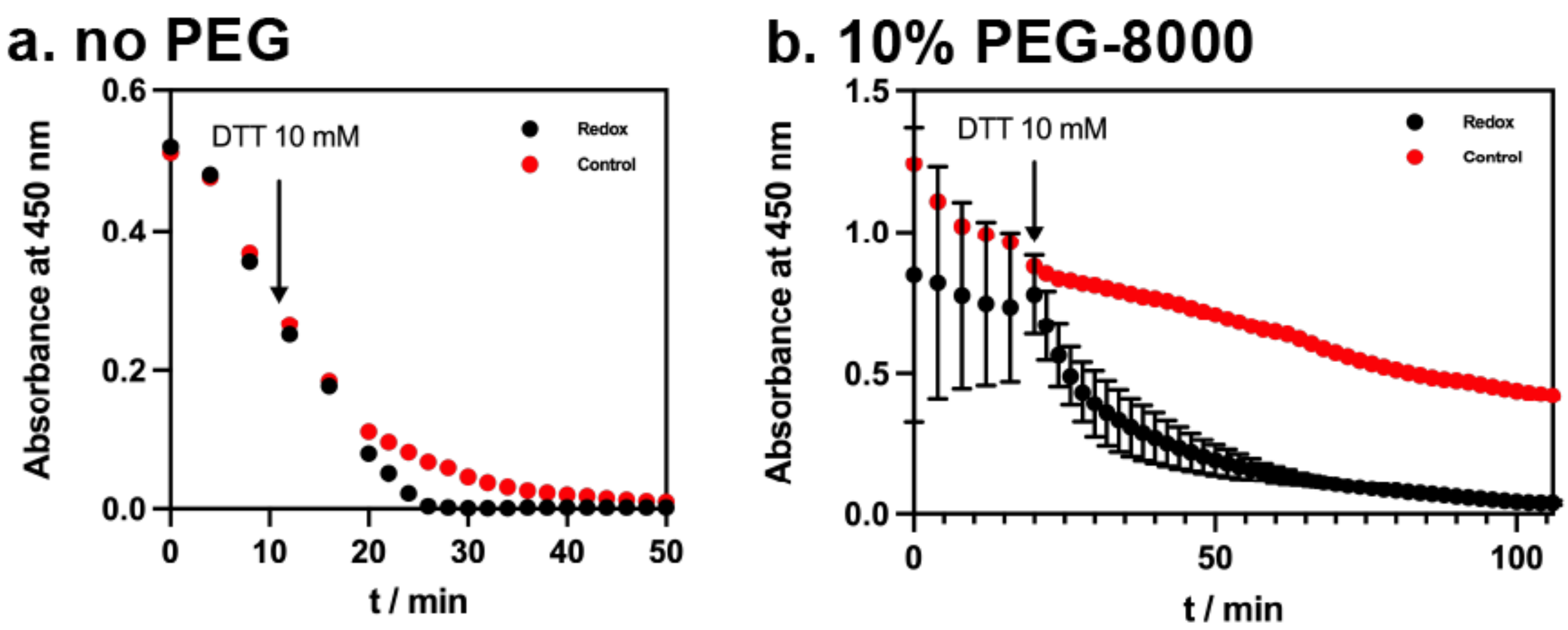

Figure S22. Turbidity measurements over time of condensates formed with **(AG)$_5$** (1 mg mL^{-1}) and **(GFRGGRC)$_2$** (1 mg mL^{-1}) (a) without polyethylene glycol 8000 (PEG-8000) and (b) with PEG-8000 (10%, w/v, right) in sodium phosphate buffer (pH 7, 0.1 M). In both figures, the black data point represents samples treated with reducing agent D,L-dithiothreitol (DTT, 10 mM) after some time, as indicated by the arrow. The red data points represent the control data, where no reducing agent was added.

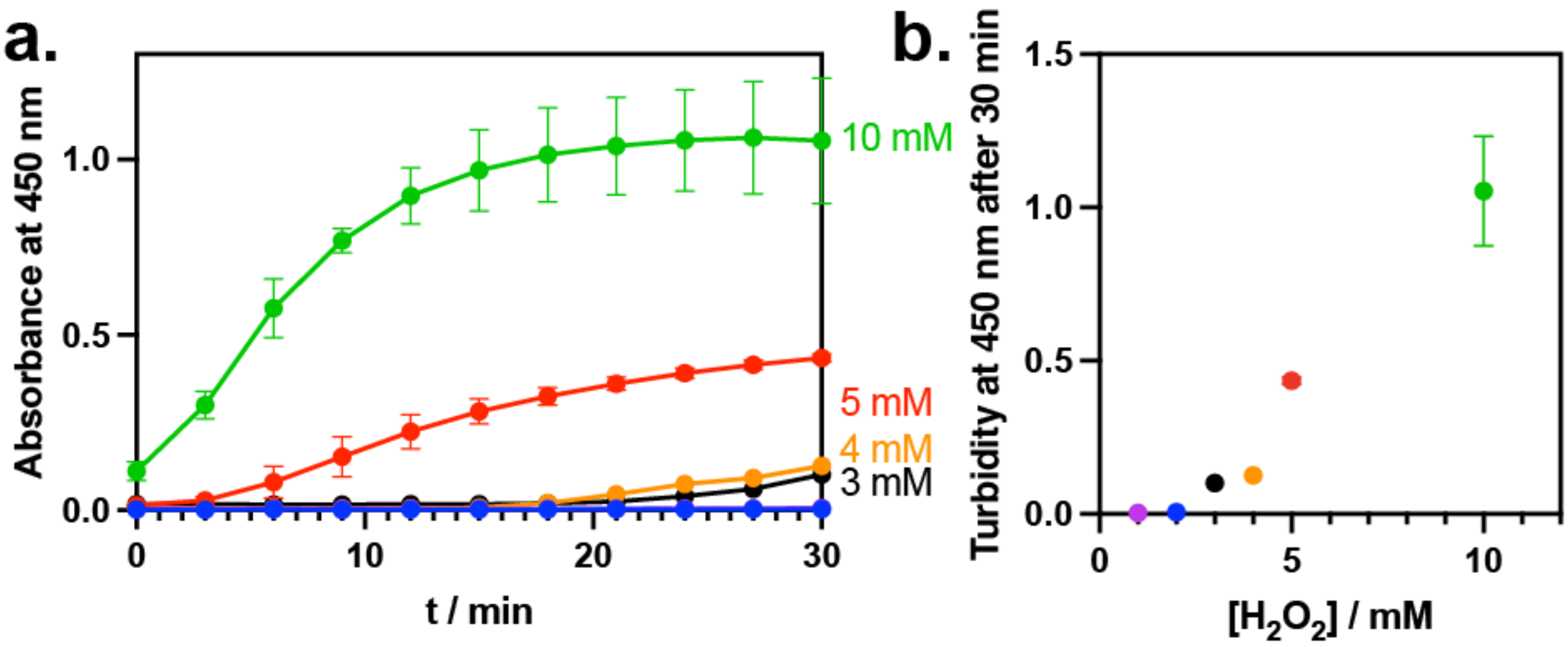

Figure S23. (a) Turbidity of solutions made with **GFRGGRC** (1 mg mL^{-1}) and **(AG)$_5$** (1 mg mL^{-1}) in sodium phosphate buffer (pH 7, 0.1 M) with PEG-8000 (10%, w/v). Hydrogen peroxide was added just before measurement, at 1 mM (purple), 2 mM (blue), 3 mM (black), 4 mM (gold), 5 mM (red) and 10 mM (green). Note that each data point was. (b) Turbidity at 450 nm of each concentration of peroxide added to **GFRGGRC** (1 mg mL^{-1}) and **(AG)$_5$** (1 mg mL^{-1}) after incubation for 30 min (endpoints of figure (a)).

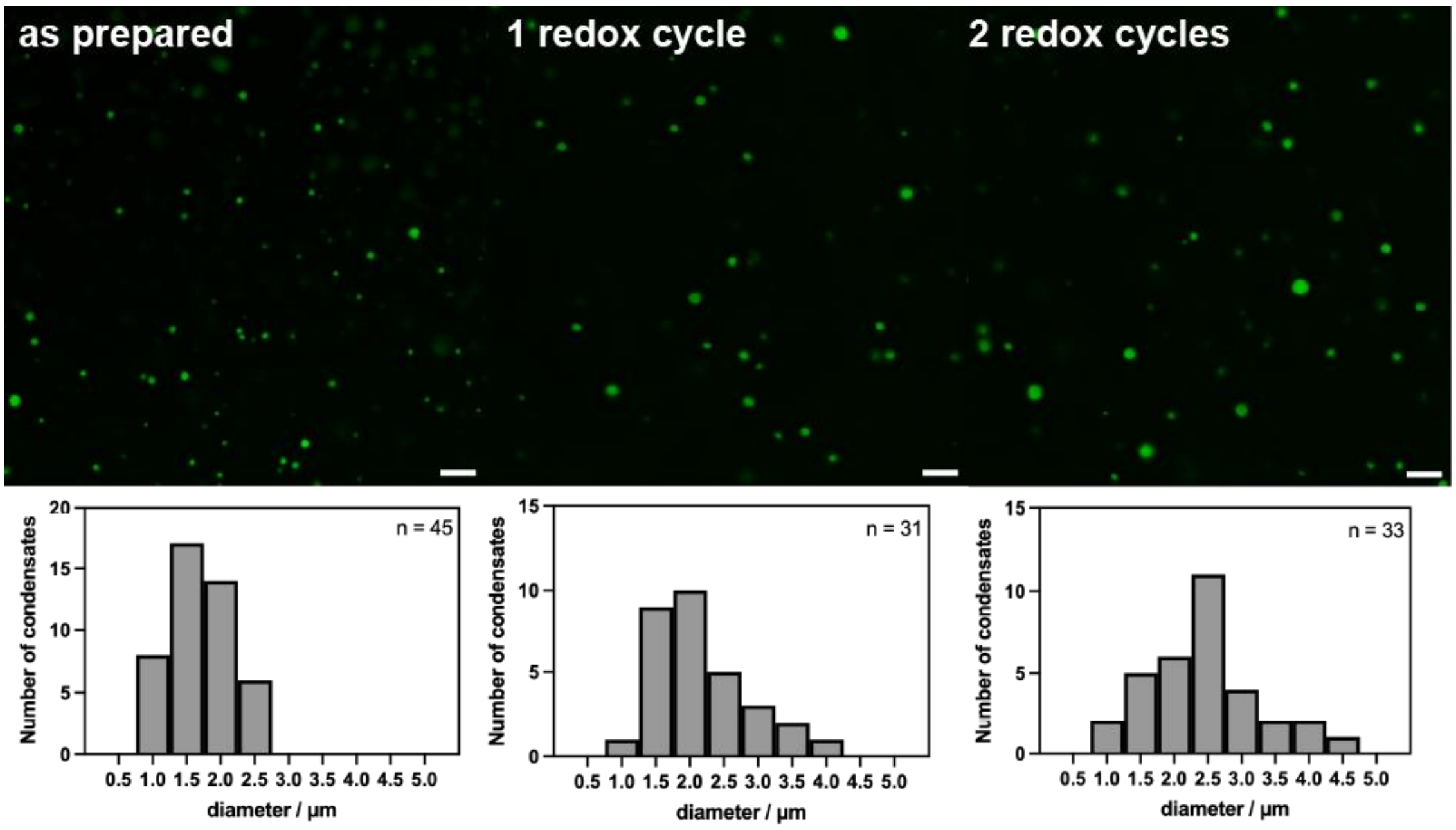

Figure S24. Confocal images of condensates formed with **GFRGGRC** (1 mg mL^{-1}, with 5% w/w **GFRGGRC-Fluor**) with **(AG)$_5$** (1 mg mL^{-1}) in sodium phosphate buffer (pH 7, 0.1 M) with PEG-8000 (10%, w/v) after multiple redox cycles and their size distributions from the image below. Each condensate in the image was measured to produce the histogram. First image was after 30 min incubation with 10 mM hydrogen peroxide, and the next two are after reduction with 10 mM DTT and oxidation with 20 mM hydrogen peroxide.

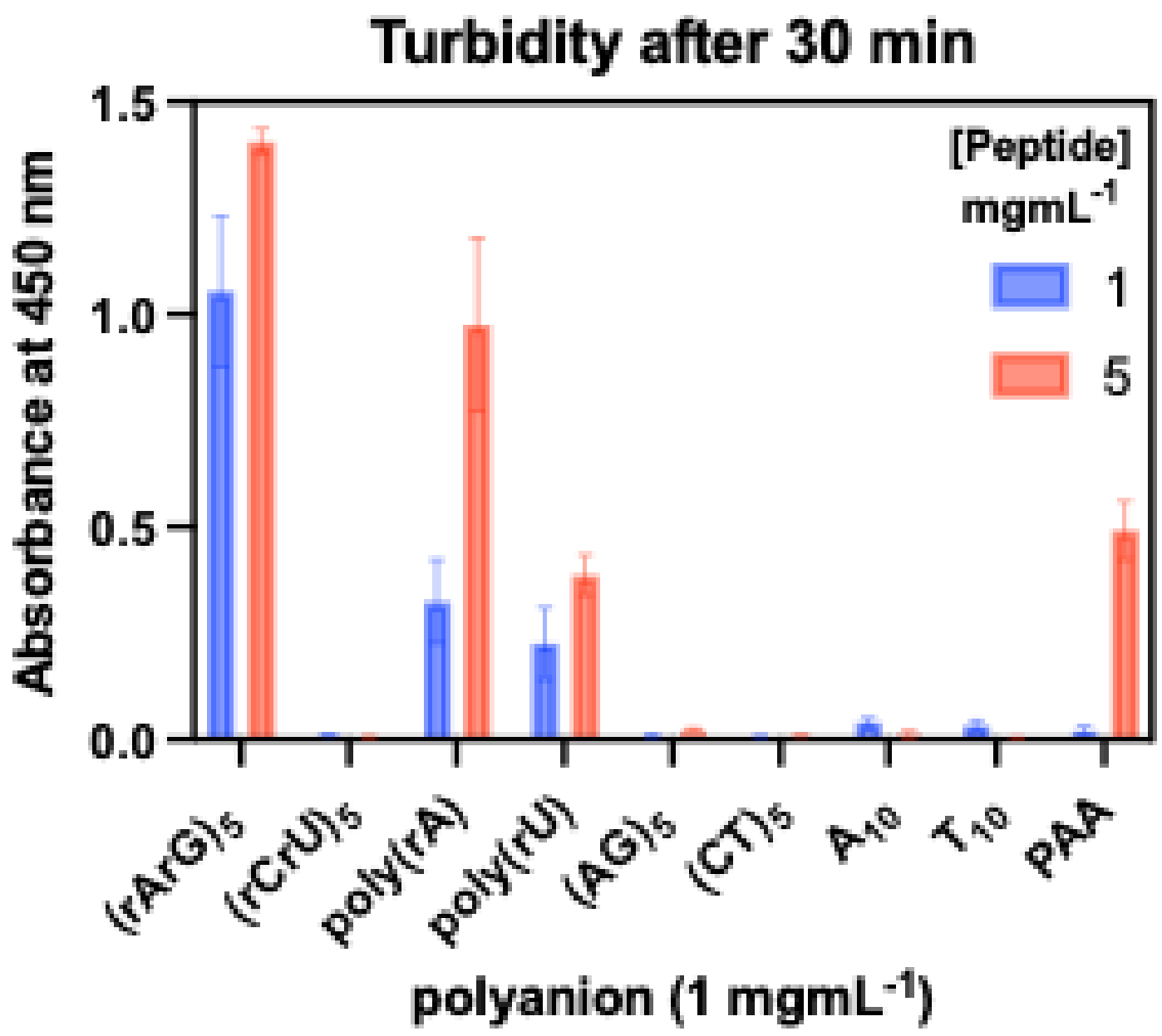

Figure S25. Absorbance at 450 nm of various polyanions (1 mg mL^{-1}) when combined with **GFRGGRC** (1 (blue) or 5 (red) mg mL^{-1}) and hydrogen peroxide (50 mM) after 30 min incubation in sodium phosphate buffer (pH 7) with PEG-8000 (10%, w/v). PAA = poly acrylic acid.

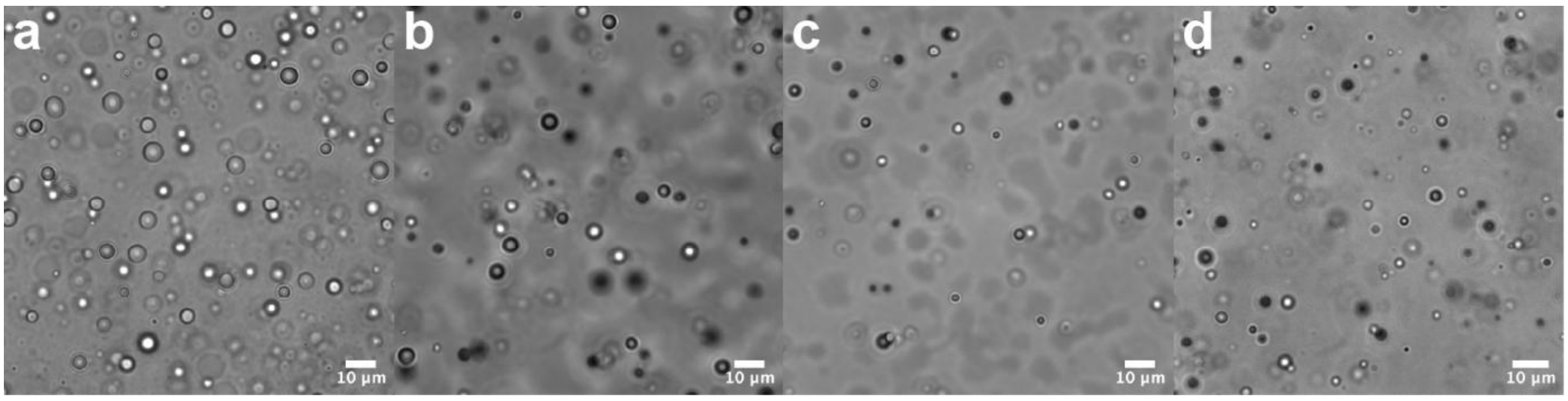

Figure S26. Brightfield microscopy images of condensates formed with **GFRGGRC** (5 mg mL^{-1}) and H_2O_2 (50 mM) with different polyanions (1 mg mL^{-1}); (a) **(AG)$_5$** RNA, (b) **poly(A)** RNA, (c) **poly(U)** RNA, and (d) poly acrylic acid in sodium phosphate buffer (pH 7) with PEG-8000 (10%, w/v).

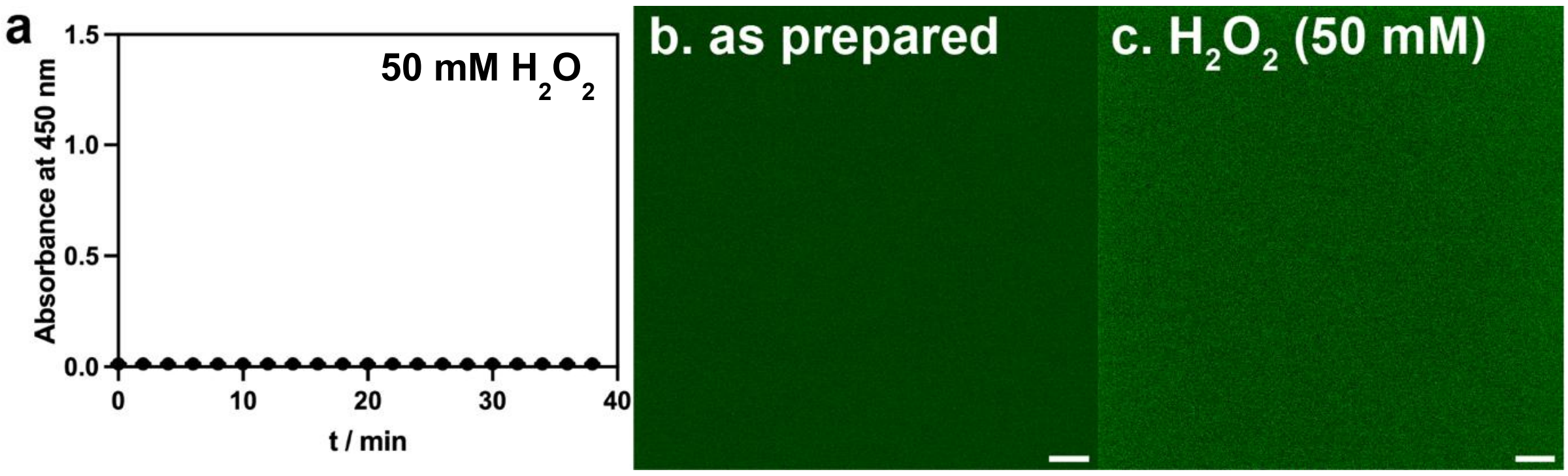

Figure S27. (a) Turbidity at 450 nm over time of **GFRGGRS** (1 mg mL^{-1}) and **(AG)$_5$** (1 mg mL^{-1}) in sodium phosphate buffer (pH 7, 0.1 M) with PEG-8000 (10%, w/v) and hydrogen peroxide (50 mM). Confocal images of (b) **GFRGGRS** (1 mg mL^{-1}, 5%, w/w **GFRGGRC-Fluor**) and **(AG)$_5$** (1 mg mL^{-1}) in sodium phosphate buffer (pH 7, 0.1 M) with PEG-8000 (10%, w/v), and (c) with the addition of 50 mM hydrogen peroxide. Note that the intensity of the background in the images is quite high to show that fluorophore is in solution.

Table S2. Tabulated data for the fluorescence recovery half-lives of the condensates before and after redox switching, with paired, two-tailed t-test p value given. For each dataset, *n* = 3. Welch's t-test was performed using GraphPad Prism version 11.0.2 for MacOS, GraphPad Software, Boston, Massachusetts USA, www.graphpad.com.

Sample	Mean	SD	t-test p value
As prepared	15.26	1.82	0.0913 (ns)
After reduction and re-oxidation	11.95	1.84	
